# Comparative Analysis of CRISPR-Cas Systems in *Pseudomonas* Genomes

**DOI:** 10.3390/genes14071337

**Published:** 2023-06-25

**Authors:** Ángel Parra-Sánchez, Laura Antequera-Zambrano, Gema Martínez-Navarrete, Vanessa Zorrilla-Muñoz, José Luis Paz, Ysaias J. Alvarado, Lenin González-Paz, Eduardo Fernández

**Affiliations:** 1 Genetics and Molecular Biology Laboratory, Biology Department, Faculty of Sciences, University of Zulia, Maracaibo 4001, Venezuela; a.parra@umh.es (Á.P.-S.); antequerazl18@gmail.com (L.A.-Z.); 2 Neuroprosthesis and Visual Rehabilitation Laboratory, Bioengineering Institute, University Miguel Hernández of Elche, 03202 Elche, Spain; gema.martinezn@umh.es; 3 Bioengineering Institute, University Miguel Hernández of Elche, 03202 Elche, Spain; vzorrilla@umh.es; 4 University Institute on Gender Studies, University Carlos III of Madrid, Getafe, 28903 Madrid, Spain; 5 Academic Department of Inorganic Chemistry, Faculty of Chemistry and Chemical Engineering, National University of San Marcos, Lima 15081, Peru; jpazr@unmsm.edu.pe; 6 Laboratory of Theoretical and Experimental Biophysical Chemistry (LQBTE), Center for Molecular Biomedicine (CBM), Venezuelan Institute for Scientific Research (IVIC), Maracaibo 4001, Venezuela; alvaradoysaias@gmail.com; 7 Laboratory of Biocomputing (LB), Center for Molecular Biomedicine (CBM), Venezuelan Institute for Scientific Research (IVIC), Maracaibo 4001, Venezuela; 8 Biomedical Research Network Center (CIBER-BBN), 28029 Madrid, Spain

**Keywords:** comparative analysis, CRISPR-Cas, *Pseudomonas*, land, environmental, clinical

## Abstract

*Pseudomonas* is a bacterial genus with some saprophytic species from land and others associated with opportunistic infections in humans and animals. Factors such as pathogenicity or metabolic aspects have been related to CRISPR-Cas, and in silico studies into it have focused more on the clinical and non-environmental setting. This work aimed to perform an in silico analysis of the CRISPR-Cas systems present in *Pseudomonas* genomes. It analyzed 275 complete genomic sequences of *Pseudomonas* taken from the NCBI database. CRISPR loci were obtained from CRISPRdb. The genes associated with CRISPR (*cas*) and CAS proteins, and the origin and diversity of spacer sequences, were identified and compared by BLAST. The presence of self-targeting sequences, PAMs, and the conservation of DRs were visualized using WebLogo 3.6. The CRISPR-like RNA secondary structure prediction was analyzed using RNAFold and MFold. CRISPR structures were identified in 19.6% of *Pseudomonas* species. In all, 113 typical CRISPR arrays with 18 putative *cas* were found, as were 2050 spacers, of which 52% showed homology to bacteriophages, 26% to chromosomes, and 22% to plasmids. No potential self-targeting was detected within the CRISPR array. All the found DRs can form thermodynamically stable secondary RNA structures. The comparison of the CRISPR/Cas system can help understand the environmental adaptability of each evolutionary lineage of clinically and environmentally relevant species, providing data support for bacterial typing, traceability, analysis, and exploration of unconventional CRISPR.

## 1. Introduction

*Pseudomonas* is a genus of Gram-negative aerobic bacteria with over 180 species [1], many of which are saprophytic in terrestrial environments. More than 25 of its species are associated with opportunistic infections in humans and animals, such as *Pseudomonas aeruginosa*, *Pseudomonas fluorescens*, *Pseudomonas putida*, *Pseudomonas stutzeri*, *Pseudomonas maltophilia*, *Pseudomonas putrefaciens*, etc., while *Pseudomonas syringae* acts as a plant pathogen [2]. Due to its ubiquity, *Pseudomonas* has been isolated from both unpolluted soils and soils contaminated by chemical and xenobiotic substances, as well as from aquatic and intra-hospital environments [3,4]. It comprises a vast group of microorganisms with extensive genetic variability and significant clinical and environmental implications. Despite the global issues caused by some pathogenic species, many of these, including the same pathogens, find applications in industry, medicine, and agriculture [5,6,7].

*P. aeruginosa* is the most significant pathogen responsible for bronchopulmonary infections in individuals with cystic fibrosis (CF) [8]. However, its secondary products have broad applications [7]. Recently, factors such as pathogenicity, tolerance to physicochemical conditions, and even aspects related to metabolic and biological cycles in microorganisms have been correlated with the presence of CRISPR-Cas systems in bacteria such as *Pseudomonas* [9,10,11]. In fact, research has demonstrated that CRISPR-Cas systems regulate biofilm production and aggregation behaviors in *Pseudomonas* [4,12,13,14].

Moreover, a larger number of initially undetected CRISPR structures may coexist in the genomes of species inhabiting both clinical and environmental settings [9]. This is due to inadequate preprocessing of microbial genomes in databases during the search for CRISPR structures. Therefore, it is crucial to employ tools that do not overlook questionable or small CRISPR structures, including those that deviate slightly from canonical structures [15].

So far, numerous studies have been conducted describing the CRISPR-Cas systems in *P. aeruginosa*, both at the structural and functional levels [16,17,18,19,20,21,22,23], but not throughout the entire *Pseudomonas* genus. The search for CRISPR structures in the genomes of the *Pseudomonas* genus is significant as it contributes to the study of the diversity and distribution of these genetic elements in various environments. Therefore, the objective of this research was to analyze the presence of CRISPR-Cas systems in *Pseudomonas* genomes across different environments.

## 2. Materials and Methods

### 2.1. Obtaining Genomic Sequences of Pseudomonas

The NCBI GenBank (National Center for Biotechnology Information, Bethesda, MD, USA) contains over 180 species of *Pseudomonas* with assembled genomes [24]. For our study, we only selected those genomes that were completely assembled. Out of more than 3000 genomes distributed across 185 species, only 275 had complete genomic sequences in the database, spanning across 51 species. Based on this, 275 *Pseudomonas* genomes were analyzed. The information created or provided by the U.S. government on the NCBI is in the public domain, and data from the National Library of Medicine (NLM, National Institutes of Health) web pages can be freely distributed and copied. Additional genomic sequences were obtained from specific websites compiled from the Entrez Genome Projects list (http://www.ncbi.nlm.nih.gov/genomes/lproks.cgi; accessed on 30 January 2020), the Genomes OnLine database (http://www.genomesonline.org/; accessed on 30 January 2020 [25]) or published genomic sequences. The molecular databases on the NCBI website are designed to provide comprehensive and up-to-date information accessible to the scientific community. The NCBI does not impose any restrictions on the use or distribution of the data contained therein, nor does it accept data when the sender requests restrictions on its reuse or redistribution. The CRISPR loci were obtained from the CRISPRdb database, which is constructed from publicly available genomic sequences [15].

### 2.2. Identification of CRISPR Structures

The CRISPR loci in the genomes were identified using CRISPRFinder [15]. A comparison of spacers was performed through alignment using the default parameters of the Muscle program. The similarity percentage of the spacers was calculated using the percentage_identity() function of the (Bio)perl interface, specifically the AlignIO methods in the Muscle interface, with a parameter set at 60%. To differentiate confirmed CRISPR structures from uncertain ones (small CRISPR-like structures with only two or three repeat sequences or DRs), they were ranked based on a Level of Evidence scale ranging from 1 to 4. Level 1 included small CRISPRs with three spacers or fewer, while levels 2 to 4 were assigned based on repeat and spacer similarity.

### 2.3. Identification and Comparison of CRISPR-Associated Genes (CAS) and CAS Proteins

In the *cas* gene search, the initial step involved identifying open reading frames (ORFs) with Prodigal [26]. These ORFs were subsequently analyzed using the MacSyFinder program to search for Hidden Markov Models (HMM) gene models within a library of known CAS proteins [27]. Alternatively, BLAST was employed to identify *cas* genes in the upstream and downstream sequences of CRISPR and TIGRFAM loci [28]. *Cas* types and subtypes were determined through *cas* cluster analysis using the CRISPRCas-Finder 1.1.2 program [15]. Phylogenetic trees were generated for the representative CAS core protein, specifically CAS1, using the MUSCLE algorithm based on the unweighted pairwise group method (UPGMA) [29]. In parallel, multiple sequence alignments and phylogenetic analyses were conducted using Clustal X, and dendrograms were visualized using the companion application NJ Plot [30]. The distance matrix was calculated using the Jaccard coefficient. Conservation of CAS proteins was evaluated using Geneious Global Alignment (Needleman–Wunsch) for multiple sequence alignments with default parameters [31].

### 2.4. Determination of the Origin and Diversity of Spacer Sequences

Unique spacer sequences and their origins were identified using the NCBI multiple sequence alignment program with default parameters. To find homologous sequences, all the spacers were compared to the GenBank database, requiring a minimum of 85% matches (at least 28 out of 33 matching nucleotides) [28,32]. Through BLAST analysis, spacer diversity was determined based on homology, considering spacers with at least 99% matches [31].

### 2.5. Identification of Protospacer Adjacent Motifs (PAMs) and Self-Targeting

The 20 bp sequences located upstream and downstream of protospacers were utilized to identify potential PAMs (Protospacer Adjacent Motifs), visualized using WebLogo 3.6 [33]. This approach also facilitated the search for self-targeting spacers both within and outside the CRISPR array through alignments (BLAST), specifically by selecting repeat spacers exhibiting 100% homology and identity. Alternatively, self-targeting-associated target genes located outside the CRISPR array were identified using GenBank.

### 2.6. Determination of the Conservation of Direct Repeats (DRs) and the Prediction of the RNA Secondary Structure

The prediction of CRISPR-like RNA secondary structures, including minimum free energy (MFE) formation and folding kinetics, was carried out using RNAfold with default parameters (http://rna.tbi.univie.ac.at/cgi-bin/RNAWebSuite/RNAfold.cgi, accessed on 25 April 2020 [34]). To represent the conservation of Direct Repeats (DRs), WebLogo 3.6 was employed, and dendrograms were utilized to facilitate clustering using the MUSCLE program. The proposed criteria for predicting the secondary structures of prokaryotic interference precursors (siRNA) transcribed by CRISPR loci, based on either DRs [31,32] or complete CRISPR structures [35], were compared. Thermodynamic parameters such as ΔG, ΔH, ΔS, and Tm were calculated using MFold.

### 2.7. Statistical Analysis

Descriptive statistical calculations were performed on the CRISPR loci, including percentage calculations to determine the proportion of spacers based on their origin. Pearson’s correlation coefficient was applied as a linear measure between two randomly selected quantitative variables. Student’s t-distribution was utilized as a statistical model to assess significant differences between the means of two groups ( *p* > 0.01). Additionally, a one-factor analysis of variance (ANOVA) was conducted to determine differences among the analyzed comparative parameters that consisted of more than two groups.

## 3. Results and Discussions

### 3.1. Identification of CRISPR Structures

CRISPR structures were found in 19.6% of *Pseudomonas* species (10 out of 51), corresponding to 17.5% of the analyzed genomes (48 out of 275). Among these, 81.2% belonged to the *P. aeruginosa* species (thirty-nine out of forty-eight), while the remaining 18.8% (nine out of forty-eight) were attributed to other species such as *Pseudomonas putida*, *stutzeri*, *chlororaphis*, *mendocina*, *pseudoalcaligenes*, *alcaliphila*, *balearica*, *parafulva*, and *citronellolis.* The origin of the strains was identified as either clinical or environmental. Out of the total, twenty-nine out of forty-eight were of clinical origin, fourteen out of forty-eight were of environmental origin, and five out of forty-eight had no specified origin according to information in the GenBank database (Table S1).

A total of 113 typical CRISPR sets were identified across the 48 genomes analyzed, averaging two CRISPR loci per genome (Table S1). These CRISPR loci were designated as CRISPR1, CRISPR2, CRISPR3, and CRISPR4 based on their relative position in the chromosome. They corresponded to types I-F, I-E, I-C, and IV of Class 1, following the classification by Mohanraju in 2016 and Makarova in 2020 [36,37], as well as the characterization by Hidalgo and Barrangou in 2020 [38].

These findings are consistent with previous literature reports, as Class 1 has been identified as the most widespread in nature [39,40], encompassing a diverse array of CAS proteins [41,42,43]. Moreover, these results align with studies conducted on *P. aeruginosa*, where the primary CRISPR types observed are IF and IE [12,13,14,16].

In this study, the length of CRISPRs exhibited significant variation, ranging from 333 to 4179 bp (both values considered outliers), with an average length of approximately 1078 bp. The number of spacers also varied, ranging from five to forty-three, with an average of seventeen spacers per CRISPR structure. The average length of individual spacers was 32 bp, allowing for interspersed direct repeats (DRs) within the range of 27 to 32 bp, as previously described [15,31,44] (Table S1).

A weak negative correlation (R^2^ = −0.23) was observed between the number of CRISPR loci and strain origin (clinical or environmental), which contradicted the expected hypothesis that environmental species would have more CRISPR loci (*p* > 0.01). Theoretically, bacteria in environmental settings are more exposed to lytic and lysogenic phages [9,43]. Additionally, no correlation was found between the number of spacers and strain origin (R^2^ = 0.03). In our study conditions, there was no statistically significant evidence to suggest that strains of clinical or environmental origin exhibited a preference for any specific CRISPR type or subtype (*p* > 0.01), and no correlation was observed between these variables (R^2^ = 0.22). Our results are consistent with the findings reported by Lyons et al. in 2015, who determined in the genus *Enterococcus* that, although habitat differences exert variable selective pressure, the incidence and distribution of CRISPR-Cas system types depend on the species [9].

### 3.2. Identification and Comparison of CRISPR-Associated Genes (cas) and CAS Proteins

Eighteen putative *cas* genes were identified upstream of the CRISPR region in Pseudomonas genomes. In all the analyzed genomes with defined CRISPR structures, CRISPR-associated genes were found, including all the genes belonging to the cas core (cas1, cas2, cas3, cas4), except in *P. putida* KF715. Various subtype genes characteristic of the csy, cse, and csf families were also present. All detected cas genes belonged to Class 1 and were distributed as shown in Table 1, as reported in other research works [45,46].

**Figure 1:**
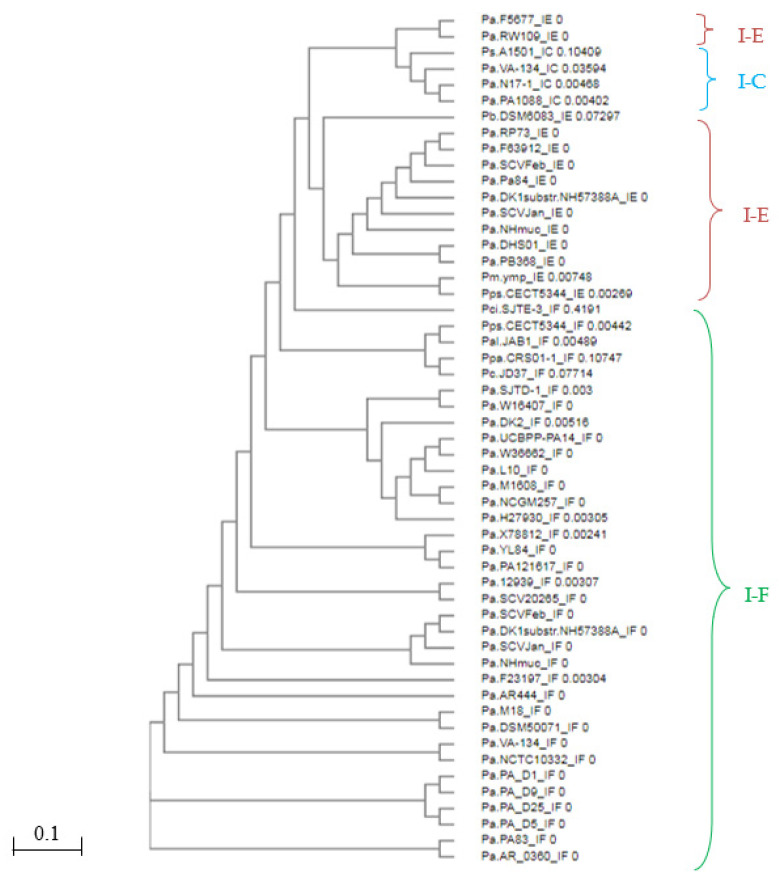
Phylogenetic tree for CAS1 proteins in *Pseudomonas* genomes. The UPGMA tree of the CAS1 protein was generated using the MUSCLE algorithm in MEGA6. Representative CAS1 proteins of the found subtypes (I-F, I-E, and I-C) were selected, excluding species *P. putida* KF715 because the cited protein was not found in its genome.

**Figure 2:**
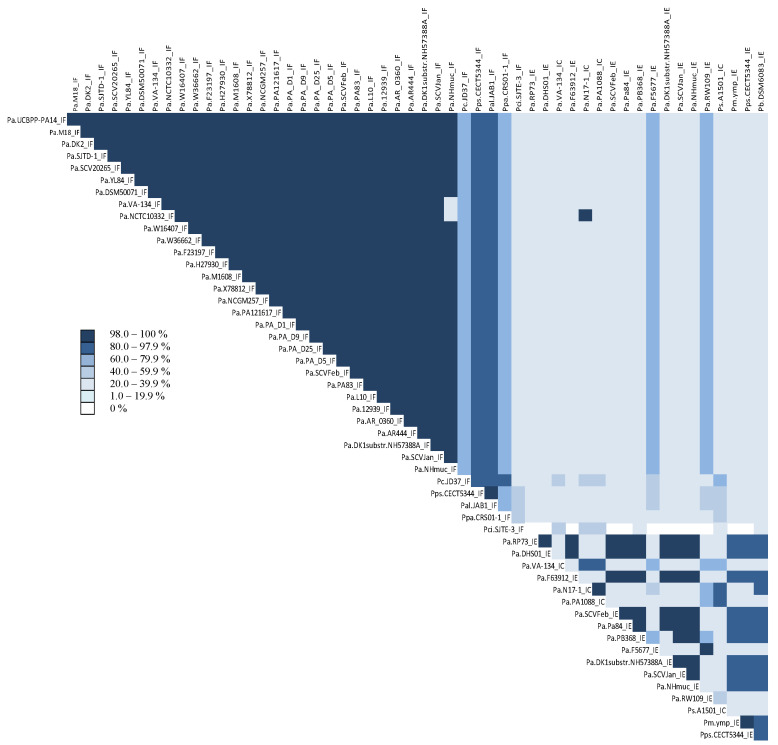
Percentage of identity of CAS1 proteins in *Pseudomonas* genomes. Abbreviations Pa, Ps, Pc, Pm, Pps, Pal, Pb, Ppa, and Pci represent the abbreviations for *Pseudomonas aeruginosa*, *stutzeri*, *chlororaphis*, *mendocina*, *pseudoalcaligenes*, *alcaliphila*, *balearica*, *parafulva*, and *citronellolis*, respectively. The species *P. putida* KF715 has been excluded because the cited protein was not found in its genome.

The phylogenetic relationship based on homology between CAS1 proteins classified species into CRISPR subtypes IF, IE, and IC (Figure 1). There was a high level of identity (>98%) among CAS1 proteins belonging to the same CRISPR array type (Figure 2). However, CAS1 proteins from *P. aeruginosa* species F5677 and RW109 of subtype I-E showed lower similarity to other proteins of the same subtype and were more closely related to proteins belonging to subtype I-C. Additionally, CAS1 from *P. citronellolis* SJTE-3 (subtype IF), with the longest phylogenetic distance (0.41) within the group, did not exhibit homology with some CAS1 proteins corresponding to the IE subtype found in *P. aeruginosa* RP73, DHS01, F63912, SCVfeb, Pa84, F5677, DK1 substr. NH57388A, SCVJan, Nhmuc, RW109, *P. mendocina* ymp, and *P. pseudoalcaligenes* CECT5344 (Figure 1 and Figure 2).

These results confirm the findings reported in the literature regarding the arrangement of CAS proteins [36,37,47]. All analyzed genomes contained the core *cas* genes, with the exception of the *P. putida* KF715 genome, which lacked *cas1* and *cas2*. This observation aligns with the studies conducted by Makarova in 2015 and Pinilla in 2020 on CRISPR systems of type IV and VI, respectively. Despite the absence of *cas1* and *cas2*, which are known to play a crucial role in spacer acquisition [36,37,48], the *P. putida* KF715 genome still exhibited other CAS proteins.

Systems of this nature rely on the presence of multisubunit effector complexes. In line with this, Ozcan (2019) demonstrated effector complex formation in *Aromatoleum aromaticum* EbN1, where a unique *cas6* variant (*csf5*) was identified. This variant is responsible for generating crRNAs, which are specifically incorporated into CRISPR-ribonucleoprotein complexes. These findings highlight evolutionary connections between type IV and type I systems [45]. The biogenesis pathways of crRNAs differ across various CRISPR-Cas types. In Class 1 systems, the CAS6 protein plays a critical role in the primary processing of precursor crRNAs. This protein was observed in the *Pseudomonas* genomes analyzed in this study, specifically in CRISPR subtypes I-F and I-E. However, it is absent in subtype I-C, where it is complemented by another subunit, CAS5d [36]. Therefore, we have used the *cas1* gene for our comparison since, according to previous research, CAS1 is the most conserved protein within the entire CAS core, both in terms of representation in CRISPR-Cas loci (gene) and conservation of the amino acid sequence [49]. Additionally, the phylogeny of CAS1 generally correlates with the organization of CRISPR-Cas loci [50]. *Cas1* has been considered as the signature of the presence of CRISPR-Cas systems in a genome [51].

CAS1 has also been compared in our genomes due to its crucial role in the adaptation stage of the CRISPR-mediated immune response [52,53], and therefore, it could be expected to coevolve with CRISPR arrays [54,55].

### 3.3. Determining the Origin of the Spacer Sequences

**Figure 3:**
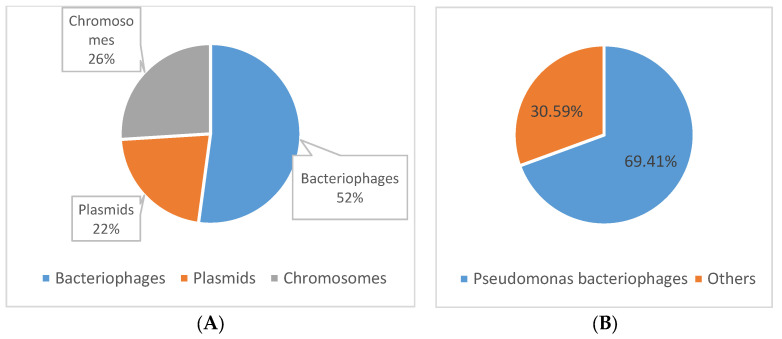
The origin of spacers. (**A**) % homology with extrachromosomal genetic material. (**B**) Comparison between the % of specific bacteriophages of *Pseudomonas* and other genera.

We identified a total of 2050 spacer sequences within the 113 detected CRISPR loci, all of which exhibited homology with sequences in the GenBank database. These spacer sequences were compared to 2303 bacteriophage sequences and 12,254 plasmid sequences. Notably, 48% of the spacers (981/2050) showed homology with extrachromosomal genetic material, with 22% associated with plasmids and 26% with chromosomes. Additionally, 52% of the spacer sequences (1069) showed homology with bacteriophages (Figure 3). Among these, 69% (742/1069) were associated with *Pseudomonas*-specific phages (Figure 3). These results suggest a potential function in immunity against foreign genetic material and indicate a high specificity of the different CRISPR-Cas systems studied. The homologies observed in the spacer sequences indicate a diverse range of recognition targets within the examined CRISPR immunity systems, encompassing both DNA sequences related to and unrelated to *Pseudomonas* representatives. Notably, the spacers demonstrated specific immunity against typical infectious bacteriophages of the *Pseudomonas* genus, including phages such as JBD25, phiCTX, phi2, phi3 F116, MP48, among others, as well as against plasmid sequences found in *Pseudomonas* strains, such as plasmid pIEC33019 or plasmid pY89. These findings underscore the prevalence of CRISPR structures in *Pseudomonas* species, providing immunity against specific lytic phages.

There was no statistically significant evidence (*p* > 0.01) to suggest that the presence of spacer sequences with homology to bacteriophages or plasmids differed significantly between clinical and environmental species. However, a moderate correlation (R^2^ = 0.41) was observed between species and a preference for spacers targeting plasmid sequences. This finding indicates that species other than the studied *P. aeruginosa*, such as *Pseudomonas putida*, *stutzeri*, *chlororaphis*, *mendocina*, *pseudoalcaligenes*, *alcaliphila*, *balearica*, *parafulva*, and *citronellolis*, exhibited a higher degree of homology in their spacer sequences with plasmids rather than bacteriophages or chromosomes.

The CRISPR-Cas system has been recognized for its role in providing immunity against viruses in prokaryotes. This system utilizes spacers derived from invading viral elements to enable the cell to mount a specific immune response by targeting homologous sequences. As a result, the profile of spacers can potentially reflect the bacterial lifestyle or habitat in which they reside [40,56].

Among the 113 observed CRISPR loci, a total of 1182 out of 2050 (57.7%) unique spacer sequences were identified. Specifically, seven hundred twenty-eight belonged to *P. aeruginosa* species, one hundred sixty-five to *P. pseudoalcaligenes*, seventy-three to *P. balearica*, fifty-one to *P. mendocina*, forty-nine to *P. parafulva*, forty-two to *P. stutzeri*, thirty-two to *P. alcaliphila*, nineteen to *P. citronellolis*, fifteen to *P. chlororaphis*, and eight to *P. putida*. Notably, except for *P. aeruginosa*, *P. mendocina*, and *P. pseudoalcaligenes*, the total number of unique spacers corresponded to the total number of spacers identified in each species (Figure S1).

The CRISPR loci discovered in *Pseudomonas* exhibited variations in length and spacer content. The longest CRISPR locus, containing sixty-eight spacers, was found in *P. balearica* DSM6083, while a second CRISPR locus in the same species had only five spacers (Figure S1). Identical CRISPR arrays were observed in different examined strains of *P. aeruginosa*: between DSM50071 and NCTC10332, between L10 and UCBPP-PA14, between PA_D1, PA_D9, PA_D25, and PA_D5, between DK1 substr. NH57388A, SCVJan, Nhmuc, and SCVfeb, and between F5677 and RW109 (Figure S1). Interestingly, these last two strains, sharing two identical CRISPR loci, had different origins. F5677 was isolated from MSKCL (Memorial Sloan Kettering Cancer Center) in New York, NY, USA, while RW109 was an industrial isolate with an undetermined locality (NCBI, http://www.ncbi.nlm.nih.gov/, accessed on 30 January 2020 [24]). Both strains are of great interest for the potential detection, biotyping, and epidemiological surveillance of these clinically and environmentally significant species [57,58].

Prokaryotic genomic sequences resemble the sequences of transmissible genetic elements such as bacteriophages and conjugative transposons [57]. This discovery has been supported by studies investigating the origin of intermediate DNA from various strains of organisms such as *Streptococcus thermophilus*, *Yersinia pestis*, or *Mycobacterium tuberculosis*, regardless of their species [59,60,61]. These findings have led to speculation that CRISPR and its associated genes may represent a form of mobile genetic element that undergoes horizontal gene transfer (HGT). This speculation is further supported by the presence of identical CRISPR loci in strains isolated from different locations, as observed in this study.

Through the amplification of prokaryotic genomes containing CRISPR and *cas* genes, it has been confirmed that HGT affects the specific *cas* genes in question. The evidence strongly suggests that this transfer likely involves the mechanism of conjugation [56,61,62,63].

### 3.4. Identification of Protospacer Adjacent Motifs (PAMs) and Self-Targeting

Experimental and in silico studies, based on CRISPR systems of some *Pseudomonas* species, have reported the recognition of PAM sequences by different protein complexes within the CRISPR-Cas system. These PAM sequences differ according to the CRISPR subtype and the species. For instance, in the CRISPR I-F subtype of *Pseudomonas aeruginosa*, the Csy complex recognizes a double-stranded GC/GC PAM [64]. In subtypes I-E and I-C of *P. aeruginosa*, the PAM sequences 5′-AAG-3′ and 5′-TTC-3′ are recognized, respectively [18,65]. In our study, no PAM sequences were identified within the analyzed CRISPR arrays of *Pseudomonas* species, providing no evidence of potential self-targeting spacers towards their own CRISPR system.

All “immune” systems must distinguish themselves from non-immune ones in order to defend against invaders without triggering autoimmunity. CRISPR loci serve to protect bacteria and archaea from the invasion of phages or DNA plasmids through a gene interference pathway. Immunity is achieved when there is a sequence match between the invading DNA and the spacers located between CRISPR repeats [66,67]. However, it is important to note that matches within the genome do not completely eliminate the possibility of spacer sequences becoming potential self-targets, thus resulting in autoimmunity.

Differential complementarity beyond the spacer sequence is an inherent feature of all CRISPR systems and plays a crucial role in the self-immunity dilemma [67]. The natural acquisition of self-targeting spacers has been observed as part of adaptive evolution studies between bacteriophages and their prokaryotic host, where only a small percentage of the observed spacers matched the genome [68], while in large-scale bioinformatics studies, these frequencies are higher. This discrepancy can be attributed in part to the selective pressure exerted by actively infecting bacteriophages [67]. One way to control the occurrence of PAM sequences within or outside the CRISPR array is by activating DNA repair mechanisms. Mutations can hinder the effectiveness of CRISPR in various ways, such as target site mutation through NHEJ, which affects spacer complementarity or PAM recognition [67].

### 3.5. Determination of the Conservation of Direct Repeats (DRs) and the Prediction of the RNA Secondary Structure

Typical CRISPR arrays with direct repeats (DRs) were identified, ranging from a minimum of six to a maximum of sixty-nine DRs, with lengths between 27 and 32 base pairs (Table S1). The number of DRs in the CRISPR loci varied across *Pseudomonas* species: *P. aeruginosa* had a range of seven to forty DRs; *P. putida* and *P. stutzeri* each had a single CRISPR structure with nine and forty-three DRs, respectively; *P. chlororaphis* had seven to ten DRs; *P. mendocina* had seventeen to thirty-nine DRs; *P. pseudoalcaligenes* had thirty-five to fifty-nine DRs; *P. alcaliphila* had fourteen to twenty DRs; *P. parafulva* had sixteen to thirty-five DRs; and *P. citronellolis* had seven to fourteen DRs. The species with the fewest and most DRs per CRISPR loci were *P. balearica* with six and sixty-nine DRs in its two CRISPR loci, respectively (Table S1).

**Figure 4:**
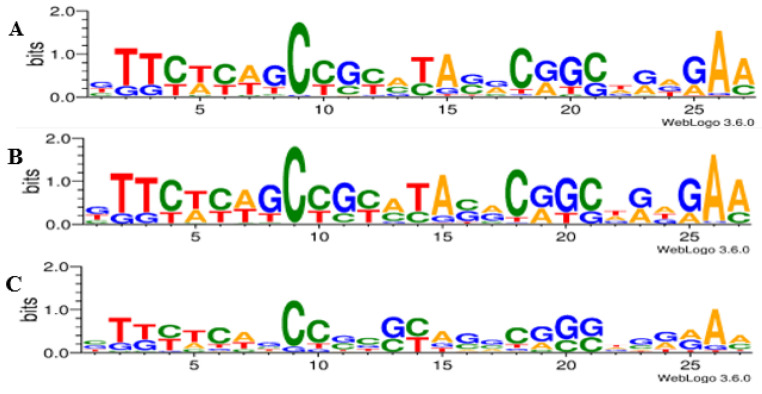
Conservation of direct repeats (DRs). The sequence logo was created by WebLogo 3.6.0. (**A**) *Pseudomonas* DRs conservation. (**B**) Conservation of the DRs of *P. aeruginosa*. (**C**) Conservation of the DRs of species other than the analyzed *P. aeruginosa*.

Multiple sequence alignments were performed to compare the CRISPR repeats. The analysis revealed a significant level of conservation in the repeat sequences (Figure 4), despite the presence of non-consensus repeats characterized by point mutations in several regions, particularly in the terminal region of the typical sequences, which resulted in the presence of non-consensus DRs (Table S2). WebLogo 3.6.0 analysis demonstrated the conservation of DRs and identified five highly conserved regions in all analyzed *Pseudomonas* genomes. These regions were located between bp 2 and 4, 9 and 11, 14 and 15, 18 and 21, and 25 and 27 of the DRs (Figure 4), with greater conservation observed among the same *P. aeruginosa* strains (Figure 4).

**Figure 5:**
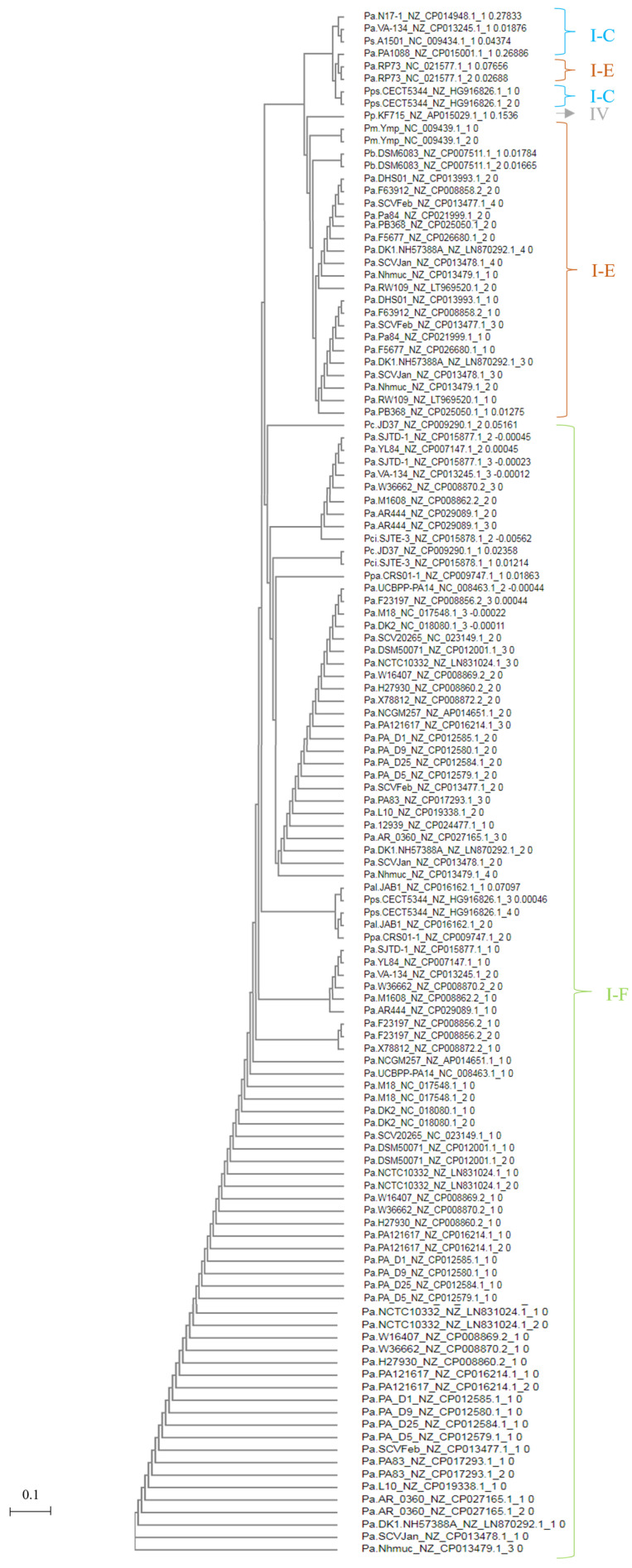
Phylogenetic tree of the consensus DRs of CRISPR loci. A total of 113 CRISPR repeat sequences were aligned using the MUSCLE algorithm in MEGA6. Coding corresponds to the *Pseudomonas* species, the NCBI code, distance, and CRISPR number.

The conservation of DRs, as depicted in Figure 5, grouped the studied species based on CRISPR subtypes (I-F, I-E, I-C, and IV). This observation indicates that families of DRs are closely associated with the gene arrangement encoding CAS proteins. As a result, the dendrogram effectively rearranged the DRs from the CRISPR loci within the same genome but with two different *cas* gene arrangements. Examples of such rearrangements can be seen in *P. aeruginosa* VA-134 (IF/IC), SCVfeb (IF/IE), SCVJan (IF-IE), Nhmuc (IF/IE), DK1 substr. NH57388A (IF/IE), and *P. pseudoalcaligenes* CECT5344 (IF/IE). This finding underscores the importance of accurately identifying CRISPR systems in databases, where the classification of CRISPR types is primarily based on DR families rather than on the arrangement of CAS proteins. Thus, it highlights the inherent close relationship between these two components [15].

The dendrogram was divided into 43 subsets. The first 31 subsets exclusively included the *P. aeruginosa* DRs with the shortest distances between them. These DRs primarily belonged to the CRISPR1 loci and entirely represented the IF subtype. The *P. aeruginosa* DRs of the same subtype, mostly found in the CRISPR2 loci, were located at level or subset 33, which was further away from the previous subsets but more closely related to the DRs of *P. pseudoalcaligenes* CECT5344 (CRISPR3 and 4), *P. alcaliphila* JAB1 (CRISPR 1 and 2), and *P. parafulva* CRS01-1 (CRISPR2). Levels 38 to 41 corresponded entirely to the DRs of species belonging to the CRISPR IE subtype, including some *P. aeruginosa*, *P. balearica* DSM6083, and *Pseudomonas mendocina ymp*. However, the DRs of the only two CRISPR loci of *P. aeruginosa* RP73 were more distantly related to this group but closely linked to the DRs of CRISPR1 and the two loci of *P. pseudoalcaligenes* CECT5344, as well as the single CRISPR locus of *P. putida* KF715, which belonged to the same subset 43 but to subtypes IC and IV, respectively. The DRs located furthest from the overall pattern and placed at the last level were those belonging to the unique CRISPR loci of *P. aeruginosa* N17-1, PA1088, *P. stutzeri* A1501, and the CRISPR1 loci of *P. aeruginosa* VA-134, all corresponding to CRISPR subtype IC (Figure 5).

**Figure 6:**
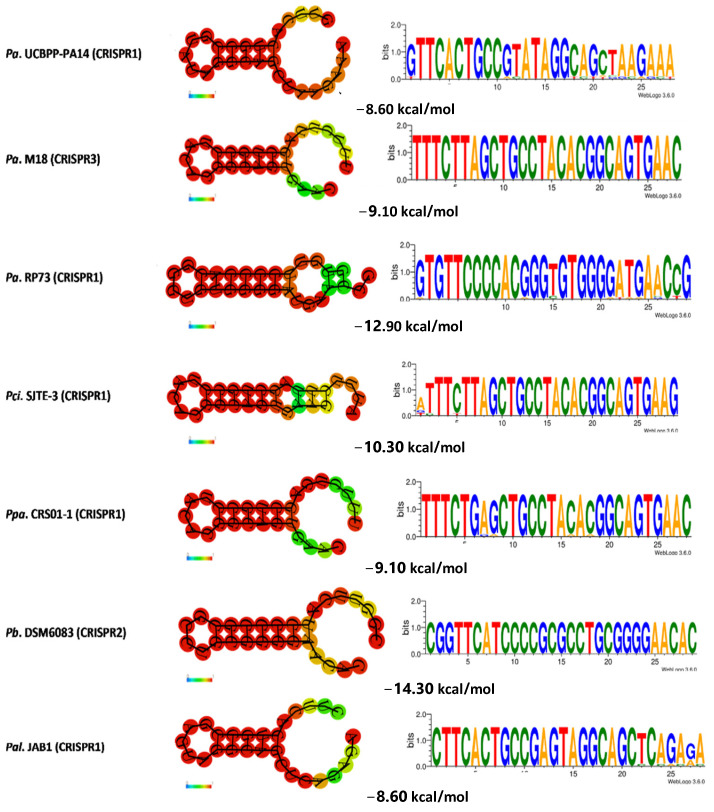
The RNA secondary structures and the minimum free energy (MFE) of formation of the direct repeats (DRs) of some found CRISPR loci and the conservation of the DRs represented with WebLogo 3.6.0. The typical stable stem-loop structures consistently predicted for DRs by RNAfold are shown. The darkest base pairs represent the highest pairing probability. Only the structures that differ are depicted.

**Figure 7:**
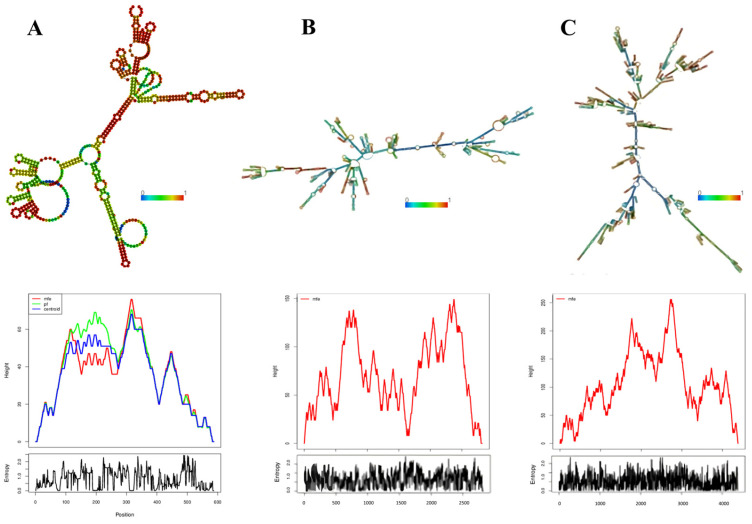
RNA secondary structures of the complete CRISPR structures (DR + spacer). Only three structures with different minimum free energy (MFEs) of formations from three species are shown. In addition, the entropy and energy levels at each position are depicted. (**A**) CRISPR2 from *P. citronellolis* SJTE-3 with an MFE of −225.30 kcal/mol. (**B**) CRISPR1 of *P. aeruginosa* PA1088 with an MFE of −1308.50 kcal/mol. (**C**) CRISPR1 from *P. balearica* DSM6083 with an MFE of −2235.00 kcal/mol.

After predicting the secondary structure of prokaryotic RNA precursor species that played a key role in the mechanism of adaptive immunity, it was determined that, on average, all DRs were capable of forming an RNA secondary structure with a thermodynamic ensemble free energy or minimum free energy (MFE) formation of −10.14 kcal/mol. The minimum energy formation was −2.70 kcal/mol, while the maximum was −16.00 kcal/mol (Table S2 and Figure 6). Additionally, the formation energy of CRISPR-like transcribed structures, calculated by determining the MFE from the analysis of the full structure (DR + spacers), showed that complete CRISPRs had an average energy of −542.97 kcal/mol. The minimum energy formation was −191.60 kcal/mol, and the maximum was −2235.00 kcal/mol (Table S2 and Figure 7).

The secondary structure of non-consensus DRs, which contained point mutations dispersed along the typical sequences, was also evaluated. It was found that the mean MFE of these variant DRs was −9.01 kcal/mol, with a minimum of −2.10 kcal/mol and a maximum of −16.00 kcal/mol. This coincided with the maximum value obtained for the consensus DRs (Table S2). In 50% of the analyzed genomes, variant or mutant DRs were observed within the CRISPR loci. Examples include the CRISPR1 loci of *P. aeruginosa* DSM50071, M1608, H27930, X78812, and 12939; the CRISPR2 loci of *P. aeruginosa* UCBPP-PA14, DHS01, SCV20265, VA-134, NCGM257, PA_D1, PA_D9, PA_D25, PA_D5, L10, PB368, and *P. balearica* DSM6083; and the CRISPR3 loci of *P. aeruginosa* AR_0360, W36662, M18, DK2, SJTD-1, NCTC10332, and PA12167 (Table S2).

**Figure 8:**
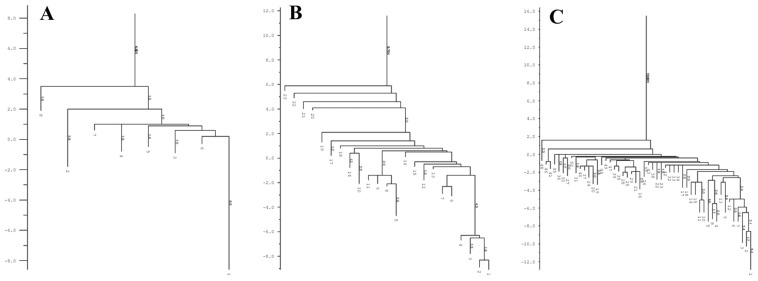
DR folding kinetics. Only three structures with different conformations are shown. (**A**) *P. alcaliphila* JAB1 CRISPR1 variant DR with a folding kinetics of 184. (**B**) *P. aeruginosa* UCBPP-PA14 CRISPR2 consensus DR with a folding kinetics of 1084. (**C**) *P. aeruginosa* CRISPR1 consensus DR RP73 with a folding kinetics of 4020.

Once transcribed, CRISPR repeats can adopt various conformations before attaining the most thermodynamically stable one (Figure 8). Therefore, when analyzing the folding kinetics of DRs, the following observations were made: an average of 1003 thermodynamically probable forms for the consensus DRs, with a minimum of 113 and a maximum of 4020; and an average of 1026 conformations for variant or mutated DRs, with a minimum of 121 and a maximum of 3921 (Table S2).

All the identified DRs can form secondary RNA structures with highly spontaneous thermodynamic stability. Several studies have predicted the thermodynamic stability of CRISPR-type transcribed structures by calculating the minimum free energy (MFE) using either the DR [43,62,69] or the complete CRISPR structure (DR + spacers) [43,69]. Both variants were considered in this analysis.

The analyses revealed a statistically significant difference (*p* < 0.01) in the minimum free energy (MFE) of assembly between the consensus and mutated DRs. This indicates that point mutations found in different regions of the repeated sequences result in significant differences in the thermodynamic mean MFE for the formation of the typical DR and its variants. The MFE increase was more pronounced in DRs where at least five base pairs of the sequence varied. Although SNPs in the sequences do not influence folding kinetics, there were no significant differences observed (*p* > 0.01).

Previous studies have reported that structures with longer stems tend to have lower MFEs and greater stability [43,69]. This stability can be influenced by the length of the stem. Accordingly, longer stems contribute to increased stability in secondary structures. This observation is consistent with Figure 6, which depicts secondary structures with longer stems in CRISPR2 of *P. mendocina* ymp, CRISPR1 from *P. aeruginosa* RP73, CRISPR2 from *P. balearica* DSM6083, and *P. stutzeri* A1501. These structures exhibited higher minimum assembly free energies of −14.20 kcal/mol, −12.90 kcal/mol, −14.30 kcal/mol, and −14.30 kcal/mol, respectively. In contrast, structures with shorter stems, such as CRISPR1 from *P. alcaliphila* JAB1, CRISPR4 from *P. pseudoalcaligenes* CECT5344, and CRISPR1 from *P. aeruginosa* UCBPP-PA14, had MFEs of −8.60 kcal/mol and were less stable compared to the aforementioned structures with higher MFEs.

**Figure 9:**
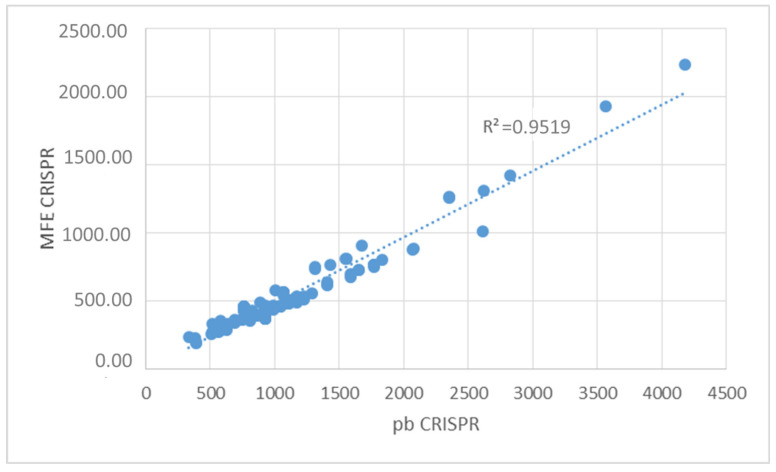
Correlation between the length (bp) of CRISPRs and the minimum free energy (MFE) of the assembly of the found CRISPRs. A very high positive correlation was evidenced (R^2^ = 0.97).

A high positive correlation (R^2^ = 0.97) was observed between the size of the CRISPR (in base pairs) and its minimum free energy (MFE). This correlation suggests that longer CRISPRs exhibit higher MFEs during formation. Consequently, the assembly of RNA secondary structures is more favorable and driven by exothermic processes (energy release) (see Figure 9).

Previous research has indicated that CRISPR repeats adopt secondary RNA structures in the form of a classic stem-loop due to their partially palindromic nature [70,71]. In the results, as shown in Figure 6, it can be observed that each CRISPR repeat exhibits one large and one small loop at both ends [72]. The majority of CRISPR loci are highly conserved, with a low occurrence of base changes in the direct repeat sequences, contributing to the overall stability of the examined full-length CRISPRs [73].

From a biophysical standpoint, it is important to understand the stability of a molecule in its different conformations, thermodynamically speaking. This could help us describe the stability of these molecular systems and determine whether they are conserved aspects or not, which would aid in understanding their distribution in various ecosystems. Understanding CRISPR biology at a transcriptional level is important because it is believed that there is a structural and functional relationship between the secondary structures derived from CRISPR arrays [72,74].

Similar to the work of Kunin et al. in 2007, our results show that stable secondary structures exhibit compensatory base changes [72]. The significant difference between different CRISPR arrays and complete CRISPR structures within the same genome may be due to some CRISPR-Cas systems having functionality outside their array, consistent with the findings of Lossada et al. in 2021. Since experimental data are lacking, evaluating the minimum free energy (MFE) of complete CRISPR structures would allow discrimination between the thermodynamics of formation for CRISPRs that have determinant roles in microorganisms and those that do not.

Based on this, it is advisable to subsequently evaluate the relationship between conserved CRISPR structures and non-conventional functions [74], as it has been described that CRISPRs associated with bioadhesion in *P. aeruginosa* have thermodynamically more stable secondary RNA structures (MFE < 0) compared to other conventional CRISPR structures within their own genome [74].

Lastly, it should be noted that one of the limitations of this study is the challenge of accurately discerning the origin of the spacers. Homology percentages between sequences originating from phages or extrachromosomal materials (from clinical or terrestrial environments) can be strikingly similar. Therefore, in order to further advance this line of research, it is crucial to conduct a comprehensive discrimination analysis to determine the origin of the spacers in terms of extrachromosomal genetic material associated with the chromosomes. This analysis would help confirm whether the sequence within the genome, which exhibits homology with the analyzed proto-spacer, corresponds to a prophage that was inserted during an infection or if it is part of a specific gene within the chromosome.

Furthermore, it will be necessary to conduct an analysis of the target genes involved in self-targeting, which encode hypothetical proteins. This analysis aims to investigate in silico whether these proteins have any functional roles, allowing us to determine if they are being suppressed by the CRISPR system.

## 4. Conclusions

This research represents the first in silico characterization report of CRISPR-Cas systems across the *Pseudomonas* genus. Bioinformatic analysis and comparison of the CRISPR/Cas system can help us understand the environmental adaptability of each evolutionary lineage of clinically and environmentally relevant species, providing data support for bacterial typing, traceability, analysis, and exploration of unconventional CRISPR.

Typical CRISPR-Cas systems were identified in the analyzed *Pseudomonas* genomes, with *P. aeruginosa* being the most abundant, representing over half of the clinically derived strains. No evidence was found linking clinical or environmental strains to specific CRISPR types or subtypes in the *Pseudomonas* genomes within the context of this study. The number or size of the CRISPR structures does not depend so much on the environment in which they are found but rather is species dependent. Strains of the same species isolated from different sites contain exactly the same CRISPR array, which is important for analyzing epidemiology and horizontal transfer.

The CRISPR arrays exhibited typical direct repeats (DRs) with a notable level of conservation in the repeated sequences, despite the presence of non-consensus repeats characterized by point mutations in different regions within each evaluated CRISPR array. Phylogenetic analysis revealed that the DRs were grouped according to their CRISPR subtype, including I-F, I-E, I-C, and IV. This indicates a close relationship between the families of DRs and the CAS protein-coding gene array types.

All identified CRISPR loci were classified as Class 1 based on the arrangement of associated *cas* genes. The majority of analyzed spacer sequences have their origin in phage-derived sequences. Less than half correspond to extrachromosomal genetic material (plasmids) and chromosomes.

There is no evidence of potential self-targeting within the analyzed CRISPR arrays, as no recognizable PAM sequences were detected by the CAS complexes. This indicates the powerful immune activity of these systems, demonstrating their active functionality.

All identified direct repeats (DR) and complete CRISPR arrays can form RNA secondary structures with negative free energies of assembly, indicating that they are thermodynamically spontaneous. Most CRISPR loci are highly conserved, contributing to the overall stability of complete CRISPR systems. The conservation of CRISPR structures enables their use in studies of the epidemiology, typing, and evolution of clinically and environmentally relevant pathogens.

## Figures and Tables

**Table 1:** CRISPR-associated genes (*cas*) type.

Subtype	*Cas* Genes	Species
IF	*cas1*, *cas2-cas3*, *cas6*, *csy1*, *csy2*, *csy3*	*P. aeruginosa* UCBPP-PA14, M18, DK2, SJTD-1, SCV20265, YL84, DSM50071, NCTC10332, W16407, W36662, F23197, H27930, M1608, X78812, NCGM2572, PA121617, PA_D1, PA_D9, PA_D25, PA_D5, PA83, L10, 12939, AR_0360, AR444. *P. chlororaphis* JD37 *P. alcaliphila* JAB1 *P. parafulva* CRS01-1 *P. citronellolis* SJTE-3
IE	*cas1*, *cas2*, *cas3*, *cas5*, *cas6*, *cse1*, *cse2*	*P. aeruginosa* RP73, DHS01, F63912, Pa84, PB368, F5677, RW109. *P. balearica* DSM6083 *P. mendocina* ymp
IC	*cas1*, *cas2*, *cas3*, *cas4*, *cas5*, *cas7*, *cas8c*	*P. aeruginosa* N17-1, PA1088, *P. stutzeri* A1501
IV	*csf1*, *csf2*, *csf3*, *csf4*, *csf5*	*P. putida* KF715
IF/IE	*cas1*, *cas2-cas3*, *cas6*, *csy1*, *csy2*, *csy3* *cas1*, *cas2*, *cas3*, *cas5*, *cas6*, *cse1*, *cse2*	*P. aeruginosa* SCVfeb, SCVJan, Nhmuc, DK1 substr. NH57388A. *P. pseudoalcaligenes* CECT5344
IF/IC	*cas1*, *cas2-cas3*, *cas6*, *csy1*, *csy2*, *csy3* *cas1*, *cas2*, *cas3*, *cas4*, *cas5*, *cas7*, *cas8c*	*P. aeruginosa* VA-134

## Data Availability

The analyzed genomes and CRISPR sequences are found, respectively, in the following databases: https://www.ncbi.nlm.nih.gov/genome/?term=Pseudomonas and https://crispr.i2bc.paris-saclay.fr/crispr/, accessed on 30 January 2020.

## Supplementary Materials

The following supporting information can be downloaded at: https://www.mdpi.com/article/10.3390/genes14071337/s1, Figure S1: Diversity of spacer sequences; Table S1: Overview of CRISPR-Cas systems in *Pseudomonas* genomes. Table S2: Thermodynamics of direct repetitions (DRs).
