# Supplementary material for: Comparative Analysis of CRISPR-Cas Systems in Pseudomonas Genomes
Source: Genes (Basel). 2023 Jun 25;14(7):1337. doi: 10.3390/genes14071337 (PMC10379622; doi:10.3390/genes14071337)
Supplement: Supplementary file 1 [file genes-14-01337-s001.zip › genes-2414244-supplementary.pdf]

TABLE S1. Overview of CRISPR-Cas systems in *Pseudomonas* genomes

Species (Strain)	Access number	CRISPR number	loci CRISPR: CRISPR length (pb)	Start	End	Spacer number	Spacer length (pb)	DR number	DR length (pb)	DR Consensus	Type of CRISPR	Number of genes *cas*	Origin of the Strain	Reference
Pa. UCBPP-PA14	NC_008463.1	2	873	2926520	2927393	14	32 - 38	15	28	GTTCACTGCCGTATAGGCAGCTAAGAAA	I-F	6	Clinical isolate . Massachusetts, USA	Lee et al., 2006
			1288	2935917	2937205	21	32 - 33	22	28	TTTCTTAGCTGCCTACACGGCAGTGAAC				
Pa. M18	NC_017548.1	3	987	1542032	1543019	16	32	17	28	GTTCACTGCCGTATAGGCAGCTAAGAAA	I-F	6	Sweet melon rizosphere. Shanghai, China	Wu et al., 2011
			866	2878171	2879037	14	32 - 34	15	28	GTTCACTGCCGTATAGGCAGCTAAGAAA				
			687	2887565	2888252	11	32	12	28	TTTCTTAGCTGCCTACACGGCAGTGAAC				
Pa. DK2	NC_018080.1	3	687	1398760	1399447	11	32	12	28	GTTCACTGCCGTATAGGCAGCTAAGAAA	I-F	6	Clinical isolate . Denmark	Rau et al., 2012
			568	2794688	2795256	9	32 - 33	10	28	GTTCACTGCCGTATAGGCAGCTAAGAAA				
			987	2803781	2804768	16	32	17	28	TTTCTTAGCTGCCTACACGGCAGTGAAC				
Pa. SJTD-1	NZ_CP015877.1	3	389	4466205	4466594	6	32 - 33	7	28	GTTCACTGCCGTGTAGGCAGCTAAGAAA	I-F	6	Contaminated oil. Shanghai, China	Liu et al., 2012
			807	4475118	4475925	13	32	14	28	TTTCTTAGCTGCCTATACGGCAGTGAAC				
			926	5782574	5783500	15	30 - 33	16	28	TTTCTTAGCTGCCTATACGGCAGTGAAC				
Pa. RP73	NC_021577.1	2	1310	1409002	1410312	21	32 - 33	22	29	GTGTTCCCCACGGGTGTGGGGATGAACCG	I-E	8	Clinical isolate . Milán, Italy	Jeukens et al., 2013
			1431	1419483	1420914	23	32	24	29	GTGTTCCCCACATGCGTGGGGATGAACCG				
Pa. DHS01	NZ_CP013993.1	2	761	4600982	4601743	12	32 - 33	13	29	CGGTTCATCCCCACGCATGTGGGGAACAC	I-E	8	Clinical isolate . France	Valot et al., 2014
			517	4610976	4611493	8	32 - 33	9	29	CGGTTCATCCCCACACCCGTGGGGAACAC				
Pa. SCV20265	NC_023149.1	2	747	1553838	1554585	12	32	13	28	GTTCACTGCCGTATAGGCAGCTAAGAAA	I-F	6	Clinical isolate . Germany	Eckweiler et al., 2014
			1226	3042757	3043983	20	31 - 32	21	28	TTTCTTAGCTGCCTACACGGCAGTGAAC				
Pa. YL84	NZ_CP007147.1	2	628	4416983	4417611	10	32 - 33	11	28	GTTCACTGCCGTGTAGGCAGCTAAGAAA	I-F	6	Compost. Kuala Lumpur. Malaysia	Chan et al., 2014
			509	5703185	5703694	8	30	9	30	TTTCTTAGCTGCCTATACGGCAGTGAACGA				
Pa. DSM 50071	NZ_CP012001.1	3	807	1484680	1485487	13	32	14	28	GTTCACTGCCGTATAGGCAGCTAAGAAA	I-F	6	Clinical isolate . Okinawa, Japan	Nakano et al., 2015
			1047	2773971	2775018	17	31 - 33	18	28	GTTCACTGCCGTATAGGCAGCTAAGAAA				
			927	2783544	2784471	15	32	16	28	TTTCTTAGCTGCCTACACGGCAGTGAAC				
Pa. VA-134	NZ_CP013245.1	3	889	3476459	3477348	13	33 - 36	14	32	GTCGCGCCCCGCACGGGCGCGTGGATTGAAAC	I-C / I-F	7 / 6	Clinical isolate . Massachusetts, USA	Miller et al., 2016
			567	5802543	5803110	9	32	10	28	GTTCACTGCCGTGTAGGCAGCTAAGAAA				
			1106	5811635	5812741	18	31 - 32	19	28	TTTCTTAGCTGCCTATACGGCAGTGAAC				
Pa. NCTC10332	NZ_LN831024.1	3	807	1484173	1484980	13	32	14	28	GTTCACTGCCGTATAGGCAGCTAAGAAA	I-F	6	Clinical isolate	Irvine et al., 2019
			1047	2773438	2774485	17	31 - 33	18	28	GTTCACTGCCGTATAGGCAGCTAAGAAA				
			927	2783010	2783937	15	32	16	28	TTTCTTAGCTGCCTACACGGCAGTGAAC				
Pa. W16407	NZ_CP008869.2	2	1227	3076220	3077447	20	32	21	28	GTTCACTGCCGTATAGGCAGCTAAGAAA	I-F	6	MSKCC. Nueva York, USA	Yan et al., 2017
			1587	3085972	3087559	26	32	27	28	TTTCTTAGCTGCCTACACGGCAGTGAAC				

Pa. W36662	NZ_CP008870.2	3	748	1697192	1697940	12	32 - 33	13	28	GTTCACTGCCGTATAGGCAGCTAAGAAA	I-F	6	MSKCC. Nueva York, USA	Yan et al., 2017
			567	3229320	3229887	9	32	10	28	GTTCACTGCCGTGTAGGCAGCTAAGAAA				
			1166	3238753	3239919	19	31 - 33	20	28	TTTCTTAGCTGCCTATACGGCAGTGAAC				
Pa. F23197	NZ_CP008856.2	3	1169	1512098	1513267	19	32 - 33	20	28	GTTCACTGCCGTACAGGCAGCTAAGAAA	I-F	6	MSKCC. Nueva York, USA	Yan et al., 2017
			928	2845847	2846775	15	32 - 33	16	28	GTTCACTGCCGTACAGGCAGCTAAGAAA				
			1770	2855424	2857194	29	31 - 32	30	29	ATTTCTTAGCTGCCTACACGGCAGTGAAC				
Pa. H27930	NZ_CP008860.2	2	807	1543749	1544556	13	32	14	28	GTTCACTGCCGTATAGGCAGCTAAGAAA	I-F	6	MSKCC. Nueva York, USA	Yan et al., 2017
			1830	2891553	2893383	30	32 - 33	31	28	TTTCTTAGCTGCCTACACGGCAGTGAAC				
Pa. M1608	NZ_CP008862.2	2	1107	3435789	3436896	18	32	19	28	GTTCACTGCCGTGTAGGCAGCTAAGAAA	I-F	6	MSKCC. Nueva York, USA	Yan et al., 2017
			803	3445420	3446233	13	34 - 38	14	28	TTTCTTAGCTGCCTATACGGCAGTGAAC				
Pa. X78812	NZ_CP008872.2	2	1168	1513945	1515113	19	32 - 33	20	28	GTTCACTGCCGTACAGGCAGCTAAGAAA	I-F	6	MSKCC. Nueva York, USA	Yan et al., 2017
			1647	2818098	2819745	27	32	28	28	TTTCTTAGCTGCCTACACGGCAGTGAAC				
Pa. NCGM257	NZ_AP014651.1	2	928	3194113	3195041	15	31	16	29	GTTCACTGCCGTATAGGCAGCTAAGAAAA	I-F	6	ND	-
			1227	3203555	3204782	20	32	21	28	TTTCTTAGCTGCCTACACGGCAGTGAAC				
Pa. F63912	NZ_CP008858.2	2	1553	4349943	4351496	25	32	26	29	CGGTTCATCCCCACGCATGTGGGGAACAC	I-E	8	MSKCC. Nueva York, USA	Yan et al., 2017
			1310	4360667	4361977	21	32 - 33	22	29	CGGTTCATCCCCACACCCGTGGGGAACAC				
Pa. N17-1	NZ_CP014948.1	1	1550	4503803	4505353	23	32 - 36	24	32	GTTTCAATCCACGCGCCCGTGCGGGGCGCGAC	I-C	7	Soils. Beijing, China	Sangare et al., 2014
Pa. PA121617	NZ_CP016214.1	3	927	4744466	4745393	15	32	16	28	GTTCACTGCCGTATAGGCAGCTAAGAAA	I-F	6	Clinical isolate	Wang et al., 2017
			809	6110306	6111115	13	32 - 33	14	28	GTTCACTGCCGTATAGGCAGCTAAGAAA				
			1047	6121528	6122575	17	32	18	28	TTTCTTAGCTGCCTACACGGCAGTGAAC				
Pa. PA_D1	NZ_CP012585.1	2	1767	2933845	2935612	29	32	30	28	GTTCACTGCCGTATAGGCAGCTAAGAAA	I-F	6	Clinical isolate	Wang et al., 2017
			567	2944127	2944694	9	32	10	28	TTTCTTAGCTGCCTACACGGCAGTGAAC				
Pa. PA_D9	NZ_CP012580.1	2	1767	3517540	3519307	29	32	30	28	GTTCACTGCCGTATAGGCAGCTAAGAAA	I-F	6	Clinical isolate	Wang et al., 2017
			567	3527822	3528389	9	32	10	28	TTTCTTAGCTGCCTACACGGCAGTGAAC				
Pa. PA_D25	NZ_CP012584.1	2	1767	2935079	2936846	29	32	30	28	GTTCACTGCCGTATAGGCAGCTAAGAAA	I-F	6	Clinical isolate	Wang et al., 2017
			567	2945361	2945928	9	32	10	28	TTTCTTAGCTGCCTACACGGCAGTGAAC				
Pa. PA_D5	NZ_CP012579.1	2	1767	2933844	2935611	29	32	30	28	GTTCACTGCCGTATAGGCAGCTAAGAAA	I-F	6	Clinical isolate	Wang et al., 2017
			567	2944126	2944693	9	32	10	28	TTTCTTAGCTGCCTACACGGCAGTGAAC				
Pa. PA1088	NZ_CP015001.1	1	2619	5428082	5430701	39	33 - 38	40	32	GTTTCAATCCACGCGCCCGTGCGGGGCGCGAC	I-C	7	Clinical isolate	Wang et al., 2017
Pa. SCVFeb	NZ_CP013477.1	4	922	2793712	2794634	15	29 - 32	16	28	GTTCACTGCCGTATAGGCAGCTAAGAAA	I-F / I-E	6 / 8	Clinical isolate	Irvine et al., 2019
			1106	2803159	2804265	18	31 - 32	19	28	TTTCTTAGCTGCCTACACGGCAGTGAAC				
			1065	4112483	4113548	17	32	18	29	CGGTTCATCCCCACGCATGTGGGGAACAC				
			760	4122719	4123479	12	32	13	29	CGGTTCATCCCCACACCCGTGGGGAACAC				

Pa. Pa84	NZ_CP021999.1	2	1067	4328236	4329303	17	32 - 33	18	29	CGGTTCATCCCCACGCATGTGGGGAACAC	I-E	8	Clinical isolate	Dößelmann et al., 2017
			578	4338539	4339117	9	32 - 33	10	29	CGGTTCATCCCCACACCCGTGGGGAACAC				
Pa. PA83	NZ_CP017293.1	3	927	1564552	1565479	15	32	16	28	GTTCACTGCCGTATAGGCAGCTAAGAAA	I-F	6	Clinical isolate	Dößelmann et al., 2017
			1404	3079377	3080781	23	28 - 33	24	28	GTTCACTGCCGTATAGGCAGCTAAGAAA				
			1648	3089307	3090955	27	32 - 33	28	28	TTTCTTAGCTGCCTACACGGCAGTGAAC				
Pa. L10	NZ_CP019338.1	2	813	2984027	2984840	13	32 - 38	14	28	GTTCACTGCCGTATAGGCAGCTAAGAAA	I-F	6	Clinical isolate	Dößelmann et al., 2017
			1107	2993364	2994471	18	32	19	28	TTTCTTAGCTGCCTACACGGCAGTGAAC				
Pa. 12939	NZ_CP024477.1	1	989	2841860	2842849	16	32 - 34	17	28	TTTCTTAGCTGCCTACACGGCAGTGAAC	I-F	6	ND	-
Pa. PB368	NZ_CP025050.1	2	1677	3738498	3740175	27	32 - 33	28	29	CGGTTCATCCCCACGCACGTGGGGAACAC	I-E	8	ND	-
			1065	3749409	3750474	17	32	18	29	CGGTTCATCCCCACACCCGTGGGGAACAC				
Pa. F5677	NZ_CP026680.1	2	821	4332752	4333573	13	32	14	29	CGGTTCATCCCCACGCATGTGGGGAACAC	I-E	8	MSKCC. Nueva York, USA	Yan et al., 2017
			761	4342805	4343566	12	32 - 33	13	29	CGGTTCATCCCCACACCCGTGGGGAACAC				
Pa. AR_0360	NZ_CP027165.1	3	627	285926	286553	10	32	11	28	GTTCACTGCCGTATAGGCAGCTAAGAAA	I-F	6	ND	-
			743	1651898	1652641	12	28 - 32	13	28	GTTCACTGCCGTATAGGCAGCTAAGAAA				
			1408	1661167	1662575	23	32 - 33	24	28	TTTCTTAGCTGCCTACACGGCAGTGAAC				
Pa. AR444	NZ_CP029089.1	3	1588	2281839	2283427	26	32 - 33	27	28	GTTCACTGCCGTGTAGGCAGCTAAGAAA	I-F	6	ND	-
			1404	2291953	2293357	23	28 - 33	24	28	TTTCTTAGCTGCCTATACGGCAGTGAAC				
			927	3805927	3806854	15	32	16	28	TTTCTTAGCTGCCTATACGGCAGTGAAC				
Pa. DK1 substr.	NZ_LN870292.1	4	922	2795174	2796096	15	29 - 32	16	28	GTTCACTGCCGTATAGGCAGCTAAGAAA	I-F / I-E	6 / 8	Clinical isolate . Denmark	Norman et al., 2016
NH57388A			1106	2804621	2805727	18	31 - 32	19	28	TTTCTTAGCTGCCTACACGGCAGTGAAC				
			1065	4113945	4115010	17	32	18	29	CGGTTCATCCCCACGCATGTGGGGAACAC				
			760	4124181	4124941	12	32	13	29	CGGTTCATCCCCACACCCGTGGGGAACAC				
Pa. SCVJan	NZ_CP013478.1	4	922	2793712	2794634	15	29 - 32	16	28	GTTCACTGCCGTATAGGCAGCTAAGAAA	I-F / I-E	6 / 8	Clinical isolate	Irvine et al., 2019
			1106	2803159	2804265	18	31 - 32	19	28	TTTCTTAGCTGCCTACACGGCAGTGAAC				
			1065	4112486	4113551	17	32	18	29	CGGTTCATCCCCACGCATGTGGGGAACAC				
			760	4122722	4123482	12	32	13	29	CGGTTCATCCCCACACCCGTGGGGAACAC				
Pa. Nhmuc	NZ_CP013479.1	4	760	1812040	1812800	12	32	13	29	CGGTTCATCCCCACACCCGTGGGGAACAC	I-E / I-F	8 / 6	Clinical isolate	Irvine et al., 2019
			1065	1821971	1823036	17	32	18	29	CGGTTCATCCCCACGCATGTGGGGAACAC				
			922	2793712	2794634	15	29 - 32	16	28	GTTCACTGCCGTATAGGCAGCTAAGAAA				
			1106	3131254	3132360	18	31 - 32	19	28	TTTCTTAGCTGCCTACACGGCAGTGAAC				
Pa. RW109	NZ_LT969520.1	2	638	4575011	4575649	10	32	11	29	CGGTTCATCCCCACGCATGTGGGGAACAC	I-E	8	Industrial isolate	Green et al., 2018
			761	4584881	4585642	12	32 - 33	13	29	CGGTTCATCCCCACACCCGTGGGGAACAC				
Pp. KF715	NZ_AP015029.1	1	518	113760	114278	8	32 - 33	9	28	CCGCTCACCCCCGCGCACGCGGGAAATAC	IV	5	Soils. AIST, Japan	Furukawa et al., 1986
Ps. A1501	NC_009434.1	1	2823	4056654	4059477	42	33 - 38	43	32	GTCGCGCCCCACGCGGGCGCGTGGATTGAAAC	I-C	7	Rizosphere. Beijing, China	Yan et al., 2008

Pc. JD37	NZ_CP009290.1	2	567	1346863	1347430	9	32	10	28	TTTCTAAGCTGCCTACACGGCAGTGAAG	I-F	6	Soils	Morohoshi et al., 2017
			388	5675857	5676245	6	31	7	28	TTTTCTAAGCTGCCTATTCGGCAGTGAAC				
Pm. Ymp	NC_009439.1	2	2351	4137496	4139847	38	31 - 33	39	29	CGGTTCATCCCCGCTGGCGCGGGGAACAC	I-E	8	Soils. Taiwan	Kao et al., 2005
			1005	4149157	4150162	16	32 - 33	17	29	CGGTTCATCCCCGCTGGCGCGGGGAACAC				
Pps. CECT5344	NZ_HG916826.1	4	2349	830038	832387	38	32 - 33	39	29	GTGTTCCCCGCGCCAGCGGGGATGAACCG	I-E / I-F	8 / 6	Guadalquir river	Garrido et al., 2018
			3567	842637	846204	58	32 - 33	59	29	GTGTTCCCCGCGCCAGCGGGGATGAACCG				
			2608	4439047	4441655	43	32 - 33	44	28	CTTCACTGCCGTGTAGGCAGCTCAGAAA				
			2067	4455183	4457250	34	32	35	28	GTTCACTGCCGTGTAGGCAGCTCAGAAA				
Pal. JAB1	NZ_CP016162.1	2	807	5187599	5188406	13	32	14	28	CTTCACTGCCGAGTAGGCAGCTCAGAGA	I-F	6	Soils. Japan	-
			1168	5199361	5200529	19	32 - 33	20	28	GTTCACTGCCGTGTAGGCAGCTCAGAAA				
Pb. DSM6083	NZ_CP007511.1	2	4179	3797904	3802083	68	32 - 33	69	29	CGGTTCATCCCCGCGCCTGCGGGGAACGC	I-E	8	Sewage water. Spain	Bennasar et al., 2016
			333	3812113	3812446	5	32	6	29	CGGTTCATCCCCGCGCCTGCGGGGAACAC				
Ppa. CRS01-1	NZ_CP009747.1	2	929	221721	222650	15	32 - 33	16	28	TTTCTGAGCTGCCTACACGGCAGTGAAC	I-F	6	Rice field	Liu et al., 2015
			2072	231406	233478	34	32 - 33	35	28	GTTCACTGCCGTGTAGGCAGCTCAGAAA				
Pci. SJTE-3	NZ_CP015878.1	2	810	204033	204843	13	31 - 32	14	29	ATTTCTTAGCTGCCTACACGGCAGTGAAG	I-F	6	Sewage water. China	Zheng et al., 2016
			386	213808	214194	6	33	7	27	TTTCTTAGCTGCCTATACGGCAGTGAA				

Pa, Pp, Ps, Pc, Pm, Pps, Pal, Pb, Ppa and *Pci* it is short for *Pseudomonas aeruginosa, putida, stutzeri, chlororaphis, mendocina, pseudoalcaligenes, alcaliphila, balearica, parafulva* and *citronellolis*, respectively. ND it is short for Undetermined.

Table S2. Thermodynamics of direct repetitions (DR).

Species (Strain)	CRISPR	Sequence type	Sequence DR (5'-3')	ΔG (kcal/mol)	ΔH (kcal/mol)	ΔS cal/(K·mol)	T_m (°C)	MFE DR (kcal/mol)	DR Folding	MFE CRISPR (kcal/mol)
***Pa.* UCBPP-PA14**	1	Consensus	GTTCACTGCCGTATAGGCAGCTAAGAAA	-8.50	-96.80	-284.7	66.8	-8.60	436	-399.10
		Variant	GTTCACTGCCGTATAGGCA**TAGGCAGCT**	-7.40	-59.10	-166.6	81.3	-7.70	801	
		Variant	**T**TTCACTGCC**AC**ATAGG**TC**G**TC**AAGAAA	-4.70	-57.50	-170.2	64.6	-2.70	762	
	2	Consensus	TTTCTTAGCTGCCTACACGGCAGTGAAC	-10.40	-68.80	-188.2	92.2	-9.10	1084	-557.80
***Pa.* M18**	1	Consensus	GTTCACTGCCGTATAGGCAGCTAAGAAA	-8.50	-96.80	-284.7	66.8	-8.60	436	-464.30
	2	Consensus	GTTCACTGCCGTATAGGCAGCTAAGAAA	-8.50	-96.80	-284.7	66.8	-8.60	436	-388.60
		Variant	GTTCACTGCCGTATAGGCAGC**A**AA**A**AA**T**	-8.40	-59.10	-163.4	88.3	-8.60	296	
		Variant	**T**TTCACTGCC**AC**ATAGG**TC**G**TC**AAGAAA	-4.70	-57.50	-170.2	64.6	-2.70	762	
	3	Consensus	TTTCTTAGCTGCCTACACGGCAGTGAAC	-10.40	-68.80	-188.2	92.2	-9.10	1084	-339.60
***Pa.* DK2**	1	Consensus	GTTCACTGCCGTATAGGCAGCTAAGAAA	-8.50	-96.80	-284.7	66.8	-8.60	436	-362.30
	2	Consensus	GTTCACTGCCGTATAGGCAGCTAAGAAA	-8.50	-96.80	-284.7	66.8	-8.60	436	-277.90
		Variant	**T**TTCACTGCC**AC**ATAGG**TC**G**TC**AAGAAA	-4.70	-57.50	-170.2	64.6	-2.70	762	
	3	Consensus	TTTCTTAGCTGCCTACACGGCAGTGAAC	-10.40	-68.80	-188.2	92.2	-9.10	1084	-437.30
***Pa.* SJTD-1**	1	Consensus	GTTCACTGCCGTGTAGGCAGCTAAGAAA	-8.50	-96.80	-284.7	66.8	-8.60	695	-198.20
	2	Consensus	TTTCTTAGCTGCCTATACGGCAGTGAAC	-10.40	-68.80	-188.2	92.2	-9.10	1117	-352.30
		Variant	TTTCTT**GA**C**GA**CCTAT**GT**GGCAGTGAA**A**	-2.20	-53.40	-165	50.3	-4.40	1535	
		Variant	TTT**T**TT**T**GCTGCCTATACGGCAGTGAAC	-11.90	-78.60	-215	92.3	-11.20	2020	
	3	Consensus	TTTCTTAGCTGCCTATACGGCAGTGAAC	-10.40	-68.80	-188.2	92.2	-9.10	1117	-421.10
***Pa.* RP73**	1	Consensus	GTGTTCCCCACGGGTGTGGGGATGAACCG	-13.40	-101.40	-283.7	84.2	-12.90	4020	-747.80
		Variant	**A**TGTTCCCCAC**A**GG**C**GTGGG**ATAA**A**C**C**T**G	-8.20	-62.00	-173.4	84.2	-8.30	1512	
	2	Consensus	GTGTTCCCCACATGCGTGGGGATGAACCG	-13.50	-101.50	-283.7	84.5	-12.90	2194	-764.50
		Variant	GTGTTCCCCACATGCGTGGGGATGAACC**A**	-13.50	-101.50	-283.7	84.5	-12.90	2168	
		Variant	GTGTTCCCCACATGCGTGGGGATGAACC**C**	-13.50	-101.50	-283.7	84.5	-12.90	1715	
***Pa.* DHS01**	1	Consensus	CGGTTCATCCCCACGCATGTGGGGAACAC	-13.00	-85.40	-233.4	92.6	-12.80	1133	-419.30
		Variant	**T**GGTTCATCCCCACGCATGTGGGGAACAC	-13.00	-85.40	-233.4	92.6	-12.80	1773	
	2	Consensus	CGGTTCATCCCCACACCCGTGGGGAACAC	-13.10	-85.50	-233.4	93.1	-12.80	1002	-332.50
***Pa.* SCV20265**	1	Consensus	GTTCACTGCCGTATAGGCAGCTAAGAAA	-8.50	-96.80	-284.7	66.8	-8.60	436	-365.20
		Variant	GTTCACTGCCGTATAGG**T**AGCTAAGAAA	-6.20	-85.80	-256.6	61.1	-6.60	511	
	2	Consensus	TTTCTTAGCTGCCTACACGGCAGTGAAC	-10.40	-68.80	-188.2	92.2	-9.10	1084	-508.60
***Pa.* YL84**	1	Consensus	GTTCACTGCCGTGTAGGCAGCTAAGAAA	-8.50	-96.80	-284.7	66.8	-8.60	695	-289.30
		Variant	**ACA**CACTGCCGTGTAGGCAGCTAAGAAA	-8.40	-59.10	-163.4	88.3	-8.60	141	
		Variant	GTTCACTGCCGTG**C**AGGCAGCTAAGAAA	-8.50	-96.80	-284.7	66.8	-8.60	590	
	2	Consensus	TTTCTTAGCTGCCTATACGGCAGTGAACGA	-10.40	-68.80	-188.2	92.2	-9.10	2082	-258.00
		Variant	TTTCTTAGCTGCCTATACGGCAGTGAAC**CC**	-10.40	-68.80	-188.2	92.2	-9.10	1137	
		Variant	TTTCTTAGCTGCCTATACGGCAGTGAACG**C**	-10.40	-68.80	-188.2	92.2	-9.10	1305	
		Variant	TTTCTTAGCTGCCTATACGGCAGTGAAC**T**A	-10.40	-68.80	-188.2	92.2	-9.10	1565	
		Variant	TTTCTTAGCTGCCTATACGGCAGTGAAC**TG**	-10.40	-68.80	-188.2	92.2	-9.10	1689	
***Pa.* DSM 50071**	1	Consensus	GTTCACTGCCGTATAGGCAGCTAAGAAA	-8.50	-96.80	-284.7	66.8	-8.60	436	-398.40
	2	Consensus	GTTCACTGCCGTATAGGCAGCTAAGAAA	-8.50	-96.80	-284.7	66.8	-8.60	436	-462.10
		Variant	GTTCACTGCCGTATAGGCAGC**A**AA**A**AA**T**	-8.40	-59.10	-163.4	88.3	-8.60	296	
		Variant	**T**TTCACTGCC**AC**ATAGG**TC**G**TC**AAGAAA	-4.70	-57.50	-170.2	64.6	-2.70	762	
	3	Consensus	TTTCTTAGCTGCCTACACGGCAGTGAAC	-10.40	-68.80	-188.2	92.2	-9.10	1084	-423.20
***Pa.* VA-134**	1	Consensus	GTCGCGCCCCGCACGGGCGCGTGGATTGAAAC	-14.00	78.30	-207.3	104.5	-14.30	3610	-488.10
		Variant	GTCGCGCCCCGCA**T**GGGCGCGTGGATTGAA**CA**	-13.70	80.50	-215.3	100.6	-14.00	3921	
		Variant	GTCGCGCCC**TA**C**G**CGGGCGCGTGGA**G**TGAAA**G**	-14.40	-81.20	-215.3	103.8	-14.60	3298	

	2	Consensus	GTTCACTGCCGTGTAGGCAGCTAAGAAA	-8.50	-96.80	-284.7	66.8	-8.60	695	-278.10
	3	Consensus	TTTCTTAGCTGCCTATACGGCAGTGAAC	-10.40	-68.80	-188.2	92.2	-9.10	1117	-501.90
		Variant	TTTCTT**GA**C**GA**CCTAT**GT**GGCAGTGAA**A**	-2.20	-53.40	-165	50.3	-4.40	1535	
		Variant	TTT**T**TT**T**GCTGCCTATACGGCAGTGAAC	-11.90	-78.60	-215	92.3	-11.20	2020	
***Pa.* NCTC10332**	1	Consensus	GTTCACTGCCGTATAGGCAGCTAAGAAA	-8.50	-96.80	-284.7	66.8	-8.60	436	-398.40
	2	Consensus	GTTCACTGCCGTATAGGCAGCTAAGAAA	-8.50	-96.80	-284.7	66.8	-8.60	436	-462.10
		Variant	GTTCACTGCCGTATAGGCAGC**A**AA**A**AA**T**	-8.40	-59.10	-163.4	88.3	-8.60	296	
		Variant	**T**TTCACTGCC**AC**ATAGG**TC**G**TC**AAGAAA	-4.70	-57.50	-170.2	64.6	-2.70	762	
	3	Consensus	TTTCTTAGCTGCCTACACGGCAGTGAAC	-10.40	-68.80	-188.2	92.2	-9.10	1084	-423.20
***Pa.* W16407**	1	Consensus	GTTCACTGCCGTATAGGCAGCTAAGAAA	-8.50	-96.80	-284.7	66.8	-8.60	436	-521.90
		Variant	GTTCACTGCCGTATAGGCAGC**C**AAGAAA	-8.50	-96.80	-284.7	66.8	-8.60	342	
		Variant	**T**TTCACTGCC**AC**ATAGG**TC**G**TC**AAGAAA	-4.70	-57.50	-170.2	64.6	-2.70	762	
	2	Consensus	TTTCTTAGCTGCCTACACGGCAGTGAAC	-10.40	-68.80	-188.2	92.2	-9.10	1084	-699.70
		Variant	TTTCTTAGCTGCCTAC**T**CGGCAGTGAAC	-10.40	-68.80	-188.2	92.2	-9.10	1054	
***Pa.* W36662**	1	Consensus	GTTCACTGCCGTATAGGCAGCTAAGAAA	-8.50	-96.80	-284.7	66.8	-8.60	436	-363.30
	2	Consensus	GTTCACTGCCGTGTAGGCAGCTAAGAAA	-8.50	-96.80	-284.7	66.8	-8.60	695	-285.70
	3	Consensus	TTTCTTAGCTGCCTATACGGCAGTGAAC	-10.40	-68.80	-188.2	92.2	-9.10	1117	-533.10
		Variant	**A**TT**T**TT**T**GCTGCCTATACGGCAGTGAAC	-11.90	-78.60	-215	92.3	-11.20	1584	
		Variant	TTTCTTAGCTGCCTATACGG**T**AGTGAAC	-8.10	-57.80	-160.2	87.5	-7.10	1586	
		Variant	TTTCTT**GA**C**GA**CCTAT**GT**GGCAGTGAA**A**	-2.20	-53.40	-165	50.3	-4.40	1535	
***Pa.* F23197**	1	Consensus	GTTCACTGCCGTACAGGCAGCTAAGAAA	-8.50	-96.80	-284.7	66.8	-8.60	384	-522.90
		Variant	GTTCACTGCCGTA**T**AGGCAGCTAAGAAA	-8.50	-96.80	-284.7	66.8	-8.60	436	
	2	Consensus	GTTCACTGCCGTACAGGCAGCTAAGAAA	-8.50	-96.80	-284.7	66.8	-8.60	384	-391.20
		Variant	GTTCACTGCCGTA**T**AGGCAGCTAAGAAA	-8.50	-96.80	-284.7	66.8	-8.60	436	
	3	Consensus	ATTTCTTAGCTGCCTACACGGCAGTGAAC	-10.40	-68.80	-188.2	92.2	-9.10	1164	-748.70
		Variant	**T**TTTCTTAGCTGCCTACACGGCAGTGAAC	-10.40	-68.80	-188.2	92.2	-9.10	1616	
***Pa.* H27930**	1	Consensus	GTTCACTGCCGTATAGGCAGCTAAGAAA	-8.50	-96.80	-284.7	66.8	-8.60	436	-373.60
	2	Consensus	TTTCTTAGCTGCCTACACGGCAGTGAAC	-10.40	-68.80	-188.2	92.2	-9.10	1084	-801.60
		Variant	TTTCTTAGCTGCCTACACGGCAG**G**GAAC	-9.20	-86.00	-247.6	74.1	-8.50	1949	
***Pa.* M1608**	1	Consensus	GTTCACTGCCGTGTAGGCAGCTAAGAAA	-8.50	-96.80	-284.7	66.8	-8.60	695	-482.70
	2	Consensus	TTTCTTAGCTGCCTATACGGCAGTGAAC	-10.40	-68.80	-188.2	92.2	-9.10	1117	375.50
		Variant	**AGCTGCCTA**TGCCTATACGGCAGTGAAC	-9.40	-68.80	-191.5	86	-8.80	469	
		Variant	TTTCTT**GA**C**GA**CCTAT**GT**GGCAGTGAA**A**	-2.20	-53.40	-165	50.3	-4.40	1535	
***Pa.* X78812**	1	Consensus	GTTCACTGCCGTACAGGCAGCTAAGAAA	-8.50	-96.80	-284.7	66.8	-8.60	384	-512.50
	2	Consensus	TTTCTTAGCTGCCTACACGGCAGTGAAC	-10.40	-68.80	-188.2	92.2	-9.10	1084	-725.30
		Variant	TTTCTTAGCTGCCTAC**T**CGGCAGTGAAC	-10.40	-68.80	-188.2	92.2	-9.10	1054	
***Pa.* NCGM257**	1	Consensus	GTTCACTGCCGTATAGGCAGCTAAGAAAA	-8.50	-96.80	-284.7	66.8	-8.60	490	-428.00
		Variant	GTTCACTGCCGTATAGGCAGCTAA**A**AAA**T**	-8.40	-59.10	-163.4	88.3	-8.60	423	
		Variant	GTTCACTGCCGTATAGGCAGCTAAGAAA**T**	-8.50	-96.80	-284.7	66.8	-8.60	611	
		Variant	GTTCACTGCCGTATAGGCAGCTA**G**GAAA**C**	-9.70	-96.80	-280.8	71.5	-8.60	888	
		Variant	**T**TTCACTGCC**AC**ATAGG**TC**G**TC**AAGAAA**C**	-4.70	-57.50	-170.2	64.6	-3.10	800	
	2	Consensus	TTTCTTAGCTGCCTACACGGCAGTGAAC	-10.40	-68.80	-188.2	92.2	-9.10	1084	-531.00
***Pa.* F63912**	1	Consensus	CGGTTCATCCCCACGCATGTGGGGAACAC	-13.00	-85.40	-233.4	92.6	-12.80	1133	-809.00
		Variant	**T**GGTTCATCCCCACGCATGTGGGGAACAC	-13.00	-85.40	-233.4	92.6	-12.80	1773	
	2	Consensus	CGGTTCATCCCCACACCCGTGGGGAACAC	-13.10	-85.50	-233.4	93.1	-12.80	1002	-731.70
		Variant	C**A**G**C**TCATCCCCAC**G**CCCGTGGGGAACAC	-13.00	-85.40	-233.4	92.6	-12.80	233	
		Variant	C**A**G**G**T**TTAT**CCCAC**G**CC**T**GTGGGGAACA**T**	-9.70	-90.20	-259.5	74.3	-9.30	1127	
***Pa.* N17-1**	1	Consensus	GTTTCAATCCACGCGCCCGTGCGGGGCGCGAC	-15.20	-88.00	-234.7	101.7	-16.00	3767	-806.50

		Variant	**C**TTTCA**C**TCCACGCGCCCG**C**G**TA**GGGCGCGAC	-15.90	-88.00	-232.4	105.3	-15.90	1251	
***Pa.* PA121617**	1	Consensus	GTTCACTGCCGTATAGGCAGCTAAGAAA	-8.50	-96.80	-284.7	66.8	-8.60	436	-424.90
	2	Consensus	GTTCACTGCCGTATAGGCAGCTAAGAAA	-8.50	-96.80	-284.7	66.8	-8.60	436	-366.60
	3	Consensus	TTTCTTAGCTGCCTACACGGCAGTGAAC	-10.40	-68.80	-188.2	92.2	-9.10	1084	-468.60
***Pa.* PA_D1**	1	Consensus	GTTCACTGCCGTATAGGCAGCTAAGAAA	-8.50	-96.80	-284.7	66.8	-8.60	436	-763.80
		Variant	**C**TTCACTGCCGTATAGGCAGCTAAGAAA	-8.80	-84.50	-244	73	-10.00	478	
		Variant	GTTCACTGCCGTATAGGCAGC**A**AA**A**AAA	-8.40	-59.10	-163.4	88.3	-8.60	258	
		Variant	**T**TTCACTGCC**AC**ATAGG**TC**G**TC**AAGAAA	-4.70	-57.50	-170.2	64.6	-2.70	762	
	2	Consensus	TTTCTTAGCTGCCTACACGGCAGTGAAC	-10.40	-68.80	-188.2	92.2	-9.10	1084	-291.80
***Pa.* PA_D9**	1	Consensus	GTTCACTGCCGTATAGGCAGCTAAGAAA	-8.50	-96.80	-284.7	66.8	-8.60	436	-763.80
		Variant	**C**TTCACTGCCGTATAGGCAGCTAAGAAA	-8.80	-84.50	-244	73	-10.00	478	
		Variant	GTTCACTGCCGTATAGGCAGC**A**AA**A**AAA	-8.40	-59.10	-163.4	88.3	-8.60	258	
		Variant	**T**TTCACTGCC**AC**ATAGG**TC**G**TC**AAGAAA	-4.70	-57.50	-170.2	64.6	-2.70	762	
	2	Consensus	TTTCTTAGCTGCCTACACGGCAGTGAAC	-10.40	-68.80	-188.2	92.2	-9.10	1084	-291.80
***Pa.* PA_D25**	1	Consensus	GTTCACTGCCGTATAGGCAGCTAAGAAA	-8.50	-96.80	-284.7	66.8	-8.60	436	-763.80
		Variant	**C**TTCACTGCCGTATAGGCAGCTAAGAAA	-8.80	-84.50	-244	73	-10.00	478	
		Variant	GTTCACTGCCGTATAGGCAGC**A**AA**A**AAA	-8.40	-59.10	-163.4	88.3	-8.60	258	
		Variant	**T**TTCACTGCC**AC**ATAGG**TC**G**TC**AAGAAA	-4.70	-57.50	-170.2	64.6	-2.70	762	
	2	Consensus	TTTCTTAGCTGCCTACACGGCAGTGAAC	-10.40	-68.80	-188.2	92.2	-9.10	1084	-291.80
***Pa.* PA_D5**	1	Consensus	GTTCACTGCCGTATAGGCAGCTAAGAAA	-8.50	-96.80	-284.7	66.8	-8.60	436	-763.80
		Variant	**C**TTCACTGCCGTATAGGCAGCTAAGAAA	-8.80	-84.50	-244	73	-10.00	478	
		Variant	GTTCACTGCCGTATAGGCAGC**A**AA**A**AAA	-8.40	-59.10	-163.4	88.3	-8.60	258	
		Variant	**T**TTCACTGCC**AC**ATAGG**TC**G**TC**AAGAAA	-4.70	-57.50	-170.2	64.6	-2.70	762	
	2	Consensus	TTTCTTAGCTGCCTACACGGCAGTGAAC	-10.40	-68.80	-188.2	92.2	-9.10	1084	-291.80
***Pa.* PA1088**	1	Consensus	GTTTCAATCCACGCGCCCGTGCGGGGCGCGAC	-15.20	-88.00	-234.7	101.7	-16.00	3767	-1308.50
		Variant	**C**TTTCA**C**TCCACGCGCCCGTGCGGGGCGCGAC	-15.20	-88.00	-234.7	101.7	-16.00	2379	
***Pa.* SCVFeb**	1	Consensus	GTTCACTGCCGTATAGGCAGCTAAGAAA	-8.50	-96.80	-284.7	66.8	-8.60	436	-368.90
		Variant	GTTCACTGCCGTATAGGCAGCTAA**A**AAA	-8.40	-59.10	-163.4	88.3	-8.60	315	
		Variant	**T**TTCACTGCC**AC**ATAGG**TC**G**TC**AAGAAA	-4.70	-57.50	-170.2	64.6	-2.70	762	
	2	Consensus	TTTCTTAGCTGCCTACACGGCAGTGAAC	-10.40	-68.80	-188.2	92.2	-9.10	1084	-503.80
		Variant	TTTCTTAGCTGCCTAC**T**CGGCAGTGAAC	-10.40	-68.80	-188.2	92.2	-9.10	1054	
	3	Consensus	CGGTTCATCCCCACGCATGTGGGGAACAC	-13.00	-85.40	-233.4	92.6	-12.80	1133	-564.60
		Variant	CGGTTCATCCCCACGCATGT**A**GGGAACAC	-7.50	-62.70	-177.9	79.1	-6.20	435	
	4	Consensus	CGGTTCATCCCCACACCCGTGGGGAACAC	-13.10	-85.50	-233.4	93.1	-12.80	1002	-456.60
		Variant	C**A**G**C**TCATCCCCAC**G**CCCGTGGGGAACAC	-13.00	-85.40	-233.4	92.6	-12.80	233	
		Variant	C**A**G**G**T**TTAT**CCCAC**G**CC**T**GTGGGGAACA**T**	-9.70	-90.20	-259.5	74.3	-9.30	1127	
***Pa.* Pa84**	1	Consensus	CGGTTCATCCCCACGCATGTGGGGAACAC	-13.00	-85.40	-233.4	92.6	-12.80	1133	-564.80
		Variant	**A**GGTTCATCCCCACGCATGTGGGGAACAC	-13.00	-85.40	-233.4	92.6	-12.80	1112	
	2	Consensus	CGGTTCATCCCCACACCCGTGGGGAACAC	-13.10	-85.50	-233.4	93.1	-12.80	1002	-356.60
		Variant	C**A**G**C**TCATCCCCAC**G**CCCGTGGGGAACAC	-13.00	-85.40	-233.4	92.6	-12.80	233	
		Variant	CGGTTCATCCCCACACCC**CGT**GGG**G**A**ACA**	-13.60	-85.50	-231.8	95.6	-12.70	1187	
		Variant	CGGTTCATCCCCACACCCGTGGGGAA**T**AC	-13.10	-85.50	-233.4	93.1	-12.80	1120	
***Pa.* PA83**	1	Consensus	GTTCACTGCCGTATAGGCAGCTAAGAAA	-8.50	-96.80	-284.7	66.8	-8.60	436	-446.70
		Variant	GTTCACTGCCGTATAGGCAG**T**TAAGAAA	-10.70	-76.80	-213.1	87.2	-9.80	682	
	2	Consensus	GTTCACTGCCGTATAGGCAGCTAAGAAA	-8.50	-96.80	-284.7	66.8	-8.60	436	-616.00
		Variant	GTTCACTGCCGTATAGGCAGCTAA**A**AAA	-8.40	-59.10	-163.4	88.3	-8.60	315	
		Variant	**T**TTCACTGCC**AC**ATAGG**TC**G**TC**AAGAAA	-4.70	-57.50	-170.2	64.6	-2.70	762	
	3	Consensus	TTTCTTAGCTGCCTACACGGCAGTGAAC	-10.40	-68.80	-188.2	92.2	-9.10	1084	-728.80

		Variant	TTTCTTAGCTGCCTACACG**A**CAGTGAAC	-2.90	-42.20	-126.7	59.8	-2.10	484	
***Pa.* L10**	1	Consensus	GTTCACTGCCGTATAGGCAGCTAAGAAA	-8.50	-96.80	-284.7	66.8	-8.60	436	-375.70
		Variant	GTTCACTGCCGTATAGGCA**TAGGCAGCT**	-7.40	-59.10	-166.6	81.3	-2.70	801	
		Variant	**T**TTCACTGCC**AC**ATAGG**TC**G**TC**AAGAAA	-4.70	-57.50	-170.2	64.6	-9.10	762	
	2	Consensus	TTTCTTAGCTGCCTACACGGCAGTGAAC	-10.40	-68.80	-188.2	92.2	-9.10	1084	-484.40
***Pa.* 12939**	1	Consensus	TTTCTTAGCTGCCTACACGGCAGTGAAC	-10.40	-68.80	-188.2	92.2	-12.80	1084	-452.40
***Pa.* PB368**	1	Consensus	CGGTTCATCCCCACGCACGTGGGGAACAC	-13.00	-85.40	-233.4	92.6	-12.80	879	-910.50
		Variant	**T**GGTTCATCCCCACGCACGTGGGGAACAC	-13.00	-85.40	-233.4	92.6	-12.80	1377	
	2	Consensus	CGGTTCATCCCCACACCCGTGGGGAACAC	-13.10	-85.50	-233.4	93.1	-12.80	1002	-537.60
***Pa.* F5677**	1	Consensus	CGGTTCATCCCCACGCATGTGGGGAACAC	-13.00	-85.40	-233.4	92.6	-12.80	1133	-428.10
		Variant	C**A**GTTCATCCCCACGCATGTGGGGAACAC	-13.00	-85.40	-233.4	92.6	-12.80	1041	
	2	Consensus	CGGTTCATCCCCACACCCGTGGGGAACAC	-13.10	-85.50	-233.4	93.1	-12.80	233	-437.00
		Variant	C**A**G**C**TCATCCCCAC**G**CCCGTGGGGAACAC	-13.00	-85.40	-233.4	92.6	-12.80	1120	
		Variant	CGGTTCATCCCCACACCCGTGGGGAA**T**AC	-13.10	-85.50	-233.4	93.1	-12.80	1120	
		Variant	**G**GGTTCATCCCCACACCCGTGGGGAACAC	-13.10	-85.50	-233.4	93.1	-12.80	1169	
		Variant	**G**GTTCA**CC**CCC**A**ACACCCGTGGGGAACAC	-8.60	-60.50	-167.3	88.3	-7.80	876	
***Pa.* AR_0360**	1	Consensus	GTTCACTGCCGTATAGGCAGCTAAGAAA	-8.50	-96.80	-284.7	66.8	-8.60	436	-311.10
		Variant	GTTCACTGCCGTATAGGCAG**T**TAAGAAA	-10.70	-76.80	-213.1	87.2	-9.80	682	
	2	Consensus	GTTCACTGCCGTATAGGCAGCTAAGAAA	-8.50	-96.80	-284.7	66.8	-8.60	436	-360.70
		Variant	GTTCACTGCCGTATAGGCAGCTAA**A**AAA	-8.40	-59.10	-163.4	88.3	-8.60	315	
		Variant	**T**TTCACTGCC**AC**ATAGG**TC**G**TC**AAGAAA	-4.70	-57.50	-170.2	64.6	-2.70	762	
	3	Consensus	TTTCTTAGCTGCCTACACGGCAGTGAAC	-10.40	-68.80	-188.2	92.2	-9.10	1084	-640.00
***Pa.* AR444**	1	Consensus	GTTCACTGCCGTGTAGGCAGCTAAGAAA	-8.50	-96.80	-284.7	66.8	-8.60	695	-676.50
		Variant	GTTCACTG**T**CGTGTAGGCAGCTAAGAAA	-5.70	-85.40	-256.9	59.1	-5.90	715	
	2	Consensus	TTTCTTAGCTGCCTATACGGCAGTGAAC	-10.40	-68.80	-188.2	92.2	-9.10	1117	-620.30
		Variant	TTTCTT**GA**C**GA**CCTAT**GT**GGCAGTGAA**A**	-2.20	-53.40	-165	50.3	-4.40	1535	
		Variant	TTT**T**TTAGCTGCCTATACGGCAGTGAAC	-10.40	-68.80	-188.2	92.2	-9.10	1538	
	3	Consensus	TTTCTTAGCTGCCTATACGGCAGTGAAC	-10.40	-68.80	-188.2	92.2	-9.10	1117	-463.90
		Variant	TTTCTTA**A**CTGCCTATACGGCAGTGAAC	-10.00	-67.90	-186.6	90.5	-9.40	868	
***Pa.* DK1 substr.**	1	Consensus	GTTCACTGCCGTATAGGCAGCTAAGAAA	-8.50	-96.80	-284.7	66.8	-8.60	436	-443.00
**NH57388A**		Variant	GTTCACTGCCGTATAGGCAGCTAA**A**AAA	-8.40	-59.10	-163.4	88.3	-8.60	315	
		Variant	**T**TTCACTGCC**AC**ATAGG**TC**G**TC**AAGAAA	-4.70	-57.50	-170.2	64.6	-2.70	762	
	2	Consensus	TTTCTTAGCTGCCTACACGGCAGTGAAC	-10.40	-68.80	-188.2	92.2	-9.10	1084	-503.80
		Variant	TTTCTTAGCTGCCTAC**T**CGGCAGTGAAC	-10.40	-68.80	-188.2	92.2	-9.10	1054	
	3	Consensus	CGGTTCATCCCCACGCATGTGGGGAACAC	-13.00	-85.40	-233.4	92.6	-12.80	1133	-564.60
		Variant	CGGTTCATCCCCACGCATGT**A**GGGAACAC	-7.50	-62.70	-177.9	79.1	-6.20	435	
	4	Consensus	CGGTTCATCCCCACACCCGTGGGGAACAC	-13.10	-85.50	-233.4	93.1	-12.80	1002	-456.60
		Variant	C**A**G**C**TCATCCCCAC**G**CCCGTGGGGAACAC	-13.00	-85.40	-233.4	92.6	-12.80	233	
		Variant	C**A**G**G**T**TTAT**CCCAC**G**CC**T**GTGGGGAACA**T**	-9.70	-90.20	-259.5	74.3	-9.30	1127	
***Pa.* SCVJan**	1	Consensus	GTTCACTGCCGTATAGGCAGCTAAGAAA	-8.50	-96.80	-284.7	66.8	-2.70	436	-368.90
		Variant	GTTCACTGCCGTATAGGCAGCTAA**A**AAA	-8.40	-59.10	-163.4	88.3	-8.60	315	
		Variant	**T**TTCACTGCC**AC**ATAGG**TC**G**TC**AAGAAA	-4.70	-57.50	-170.2	64.6	-2.70	762	
	2	Consensus	TTTCTTAGCTGCCTACACGGCAGTGAAC	-10.40	-68.80	-188.2	92.2	-9.10	1084	-503.80
		Variant	TTTCTTAGCTGCCTAC**T**CGGCAGTGAAC	-10.40	-68.80	-188.2	92.2	-9.10	1054	
	3	Consensus	CGGTTCATCCCCACGCATGTGGGGAACAC	-13.00	-85.40	-233.4	92.6	-12.80	1133	-564.60
		Variant	CGGTTCATCCCCACGCATGT**A**GGGAACAC	-7.50	-62.70	-177.9	79.1	-6.20	435	
	4	Consensus	CGGTTCATCCCCACACCCGTGGGGAACAC	-13.10	-85.50	-233.4	93.1	-12.80	1002	-456.60
		Variant	CAG**C**TCATCCCCAC**G**CCCGTGGGGAACAC	-13.00	-85.40	-233.4	92.6	-12.80	233	

		Variant	CAGGTTTATCCCACGCCTGTGGGGAACAT	-9.70	-90.20	-259.5	74.3	-9.30	1127	
***Pa.* Nhmuc**	1	Consensus	CGGTTCATCCCCACACCCGTGGGGAACAC	-13.10	-85.50	-233.4	93.1	-12.80	1002	-456.60
		Variant	CAGCTCATCCCCACGCCCGTGGGGAACAC	-13.00	-85.40	-233.4	92.6	-12.80	233	
		Variant	CAGGTTTATCCCACGCCTGTGGGGAACAT	-9.70	-90.20	-259.5	74.3	-9.30	1127	
	2	Consensus	CGGTTCATCCCCACGCATGTGGGGAACAC	-13.00	-85.40	-233.4	92.6	-12.80	1133	-564.60
		Variant	CGGTTCATCCCCACGCATGTAGGGAACAC	-7.50	-62.70	-177.9	79.1	-6.20	435	
	3	Consensus	GTTCACTGCCGTATAGGCAGCTAAGAAA	-8.50	-96.80	-284.7	66.8	-8.60	436	-368.90
		Variant	GTTCACTGCCGTATAGGCAGCTAAAAAA	-8.40	-59.10	-163.4	88.3	-2.70	315	
		Variant	TTTCACTGCCACATAGGTCGTCAAGAAA	-4.70	-57.50	-170.2	64.6	-2.70	762	
	4	Consensus	TTTCTTAGCTGCCTACACGGCAGTGAAC	-10.40	-68.80	-188.2	92.2	-9.10	1084	-503.80
		Variant	TTTCTTAGCTGCCTACTCGGCAGTGAAC	-10.40	-68.80	-188.2	92.2	-9.10	1054	
***Pa.* RW109**	1	Consensus	CGGTTCATCCCCACGCATGTGGGGAACAC	-13.00	-85.40	-233.4	92.6	-12.80	1133	-334.80
		Variant	CGGTTCATCCCCACGCATGTAGGGAACAC	-7.50	-62.70	-177.9	79.1	-6.20	435	
	2	Consensus	CGGTTCATCCCCACACCCGTGGGGAACAC	-13.10	-85.50	-233.4	93.1	-12.80	1002	-437.00
		Variant	CAGCTCATCCCCACGCCCGTGGGGAACAC	-13.00	-85.40	-233.4	92.6	-12.80	233	
		Variant	GGTTCACCCCCAACACCCGTGGGGAACAC	-8.60	-60.50	-167.3	88.3	-7.80	876	
		Variant	CGGTTCATCCCCACACCCGTGGGGAATAC	-13.10	-85.50	-233.4	93.1	-12.80	1120	
		Variant	GGGTTCATCCCCACACCCGTGGGGAACAC	-13.10	-85.50	-233.4	93.1	-12.80	1169	
***Pp.* KF715**	1	Consensus	CCGCTCACCCCCGCGCACGCGGGAAATAC	-9.60	-63.40	-173.4	92.3	-9.80	113	-275.30
		Variant	CCGCTTACCCCCGCGCACGCGGGAAATAC	-9.60	-63.40	-173.4	92.3	-9.80	251	
***Ps.* A1501**	1	Consensus	GTCGCGCCCCACGCGGGCGCGTGGATTGAAAC	-14.00	-78.30	-207.3	104.5	-14.30	3339	-1422.00
		Variant	GTCGCGCCCCACGCGGGAGCGTGGATTGAAAC	-13.00	-90.00	-248.2	89.3	-13.50	2424	
***Pc.* JD37**	1	Consensus	TTTCTAAGCTGCCTACACGGCAGTGAAG	-10.40	-68.80	-188.2	92.2	-9.90	1452	-271.70
		Variant	TTTCTAAGCTGCCTATACGGCAGTGAAG	-10.40	-68.80	-188.2	92.2	-9.90	1506	
	2	Consensus	TTTTCTAAGCTGCCTATTCGGCAGTGAAC	-10.40	-68.80	-188.2	92.2	-9.10	1534	-191.60
		Variant	CTTTCTAAGCTGCCTATCCGGCAGTGAAC	-10.40	-68.80	-188.2	92.2	-9.10	858	
		Variant	TTTTCTAAGCTGCCTATCCGGCAGTGAAC	-10.40	-68.80	-188.2	92.2	-9.10	1239	
***Pm.* Ymp**	1	Consensus	CGGTTCATCCCCGCTGGCGCGGGGAACAC	-14.10	-84.90	-228.2	98.7	-14.20	1369	-1264.90
		Variant	CGGTTCATCCCCGCTGGCGCGGGAACACG	-9.20	-61.40	-168.3	91.6	-9.70	1308	
		Variant	CTCGACATCCCCGCTGGCGCGGGGAACAC	-14.10	-84.90	-228.2	98.7	-14.20	402	
		Variant	TGGTTCATCCCCGCTGGCGCGGGGAACAC	-14.10	-84.90	-228.2	98.7	-14.20	2124	
	2	Consensus	CGGTTCATCCCCGCTGGCGCGGGGAACAC	-14.10	-84.90	-228.2	98.7	-14.20	1369	-581.50
		Variant	TTTTCCATCCCCGCATGTGCAGGGAACAC	-8.80	-67.60	-189.5	83.4	-8.10	687	
***Pps.* CECT5344**	1	Consensus	GTGTTCCCCGCGCCAGCGGGGATGAACCG	-15.10	-102.90	-283	90.3	-15.20	2248	-1256.20
		Variant	GTGTTCCCCGCGCCAGCAGGGATGAACCG	-9.40	-82.70	-236.3	76.7	-9.00	862	
		Variant	GTGTTCCCCGCGCCAGCGGGGATGAACCA	-15.10	-102.90	-283	90.3	-15.20	2199	
		Variant	GTGTTCCCCGCGCCAGCGGGGATGAACCC	-15.10	-102.90	-283	90.3	-15.20	1740	
		Variant	GTGTTCCCCGCGCCAGCGGGGATGAACCT	-15.10	-102.90	-283	90.3	-15.20	1762	
	2	Consensus	GTGTTCCCCGCGCCAGCGGGGATGAACCG	-15.10	-102.90	-283	90.3	-15.20	2248	-1930.30
		Variant	GTGTTCCCCGCGCCAGCGGGGATGAGCTT	-15.50	-108.60	-300.1	88.6	-15.90	1909	
		Variant	GTGTTCCCCGCGCCTGCGGGGATGAACCG	-14.90	-102.90	-283.7	89.5	-14.40	2802	
		Variant	GTGCTCCCCGCGCCAGCGGGGATGAACCG	-14.30	-83.80	-224	100.8	-15.10	1187	
		Variant	GTGTTCCCCGCGCCAGCGGGGATGAACCC	-15.10	-102.90	-283	90.3	-15.20	1740	
	3	Consensus	CTTCACTGCCGTGTAGGCAGCTCAGAAA	-8.80	-84.50	-244	73	-8.60	704	-1012.90
		Variant	CTTCACTGCCGTATAGGCAGCCCGCCAC	-8.40	-59.10	-163.4	88.3	-8.60	131	
		Variant	CTTCACTGCCGAGTAGGCAGCTCAGCAA	-8.40	-59.10	-163.4	88.3	-8.60	552	
	4	Consensus	GTTCACTGCCGTGTAGGCAGCTCAGAAA	-8.50	-96.80	-284.7	66.8	-8.60	681	-878.30
		Variant	GTTCACTGCCGTGTAGGCAGCAATTTGC	-8.40	-59.10	-163.4	88.3	-8.60	1268	

***Pal.* JAB1**	1	Consensus	CTTCACTGCCGAGTAGGCAGCTCAGAGA	**-8.40**	**-59.10**	**-163.4**	**88.3**	**-8.60**	**1098**	**-379.70**
		Variant	CTTCACTGCCGAGTAGGCAGC**C**C**CACAC**	**-8.40**	**-59.10**	**-163.4**	**88.3**	**-8.60**	**184**	
	2	Consensus	GTTCACTGCCGTGTAGGCAGCTCAGAAA	**-8.50**	**-96.80**	**-284.7**	**66.8**	**-8.60**	**681**	**-489.30**
		Variant	GTTCACTGCCGTGTAGGCAGC**AATTTGC**	**-8.40**	**-59.10**	**-163.4**	**88.3**	**-8.60**	**1268**	
***Pb.* DSM6083**	1	Consensus	CGGTTCATCCCCGCGCCTGCGGGGAACGC	**-14.50**	**-86.90**	**-233.4**	**99.1**	**-14.30**	**1829**	**-2235.00**
		Variant	**GAA**TTCATCCCCGCGCCTGCGGGGAACGC	**-14.50**	**-86.90**	**-233.4**	**99.1**	**-14.30**	**584**	
		Variant	**T**GGTTCATCCCCGCGCCTGCGGGGAACGC	**-14.50**	**-86.90**	**-233.4**	**99.1**	**-14.30**	**1997**	
		Variant	CGGTTCATCCCC**A**CGCCTGCGGGGAACGC	**-9.00**	**-64.80**	**-179.9**	**87**	**-8.60**	**1069**	
		Variant	CGGTTCAT**T**CCCGCGCCTGCGGGGAACGC	**-11.80**	**-70.30**	**-188.6**	**99.5**	**-12.20**	**2079**	
	2	Consensus	CGGTTCATCCCCGCGCCTGCGGGGAACAC	**-14.50**	**-86.90**	**-233.4**	**99.1**	**-14.30**	**1128**	**-232.00**
***Ppa.* CRS01-1**	1	Consensus	TTTCTGAGCTGCCTACACGGCAGTGAAC	**-10.40**	**-68.80**	**-188.2**	**92.2**	**-9.10**	**1086**	**-422.50**
		Variant	TTTCTGA**A**CTGCCTACACGGCAGTGAAC	**-10.00**	**-67.90**	**-186.6**	**90.5**	**-9.40**	**897**	
		Variant	TTTCTGAGCTGCCTA**A**ACGGCAGTGAAC	**-10.40**	**-68.80**	**-188.2**	**92.2**	**-9.10**	**919**	
		Variant	TTTCTG**G**GCTGCCTACA**A**GGCAGTGAAC	**-10.90**	**-69.00**	**-187.3**	**95.1**	**-9.90**	**1603**	
	2	Consensus	GTTCACTGCCGTGTAGGCAGCTCAGAAA	**-8.50**	**-96.80**	**-284.7**	**66.8**	**-8.60**	**681**	**-884.80**
		Variant	GTTCACTGCC**A**TGTAGGCAGCTCAGAAA	**-7.80**	**-97.70**	**-289.8**	**63.9**	**-7.80**	**546**	
		Variant	GTTCACTGCCGTGTAGGCAG**T**TCAGAAA	**-10.30**	**-89.50**	**-255.3**	**77.3**	**-9.80**	**994**	
		Variant	GTTCACTGCCGTGTAGGC**T**GCTCAGAAA	**-5.20**	**-43.70**	**-124.1**	**78.8**	**-4.50**	**844**	
		Variant	GTTCACTG**T**CGTGTAGGCAGCTCAGAAA	**-5.70**	**-85.40**	**-256.9**	**59.1**	**-5.90**	**703**	
***Pci.* SJTE-3**	1	Consensus	ATTTCTTAGCTGCCTACACGGCAGTGAAG	**-10.90**	**-77.20**	**-213.7**	**87.9**	**-10.30**	**2061**	**-416.40**
		Variant	**GC**TT**T**TTAGCTGCCTACACGGCAGTGAAG	**-10.40**	**-68.80**	**-188.2**	**92.2**	**-9.40**	**2716**	
		Variant	**G**TTTCTTAGCTGCCTACACGGCAGTGAAG	**-10.90**	**-77.20**	**-213.7**	**87.9**	**-10.30**	**2189**	
		Variant	**T**TTTCTTAGCTGCCTACACGGCAGTGAAG	**-10.90**	**-77.20**	**-213.7**	**87.9**	**-10.30**	**3104**	
	2	Consensus	TTTCTTAGCTGCCTATACGGCAGTGAA	**-10.40**	**-68.80**	**-188.2**	**92.2**	**-9.10**	**1117**	**-225.30**
		Variant	**GGGAAG**A**A**CTGCCTATACGGCAGTGAA	**-10.00**	**-67.90**	**-186.6**	**90.5**	**-9.40**	**121**	
		Variant	TTTCTTAGCTGC**T**TATACGGCAGTGAA	**-9.00**	**-66.30**	**-184.7**	**85.7**	**-6.60**	**1271**	

The values of ΔG, ΔH, ΔS and Tm which refer to the energy of Gibbs, enthalpy, entropy and melting temperature, respectively, were calculated through the MFold, while the MFE DR, folding kinetics and MFE CRISPR were calculated using RNAFold.

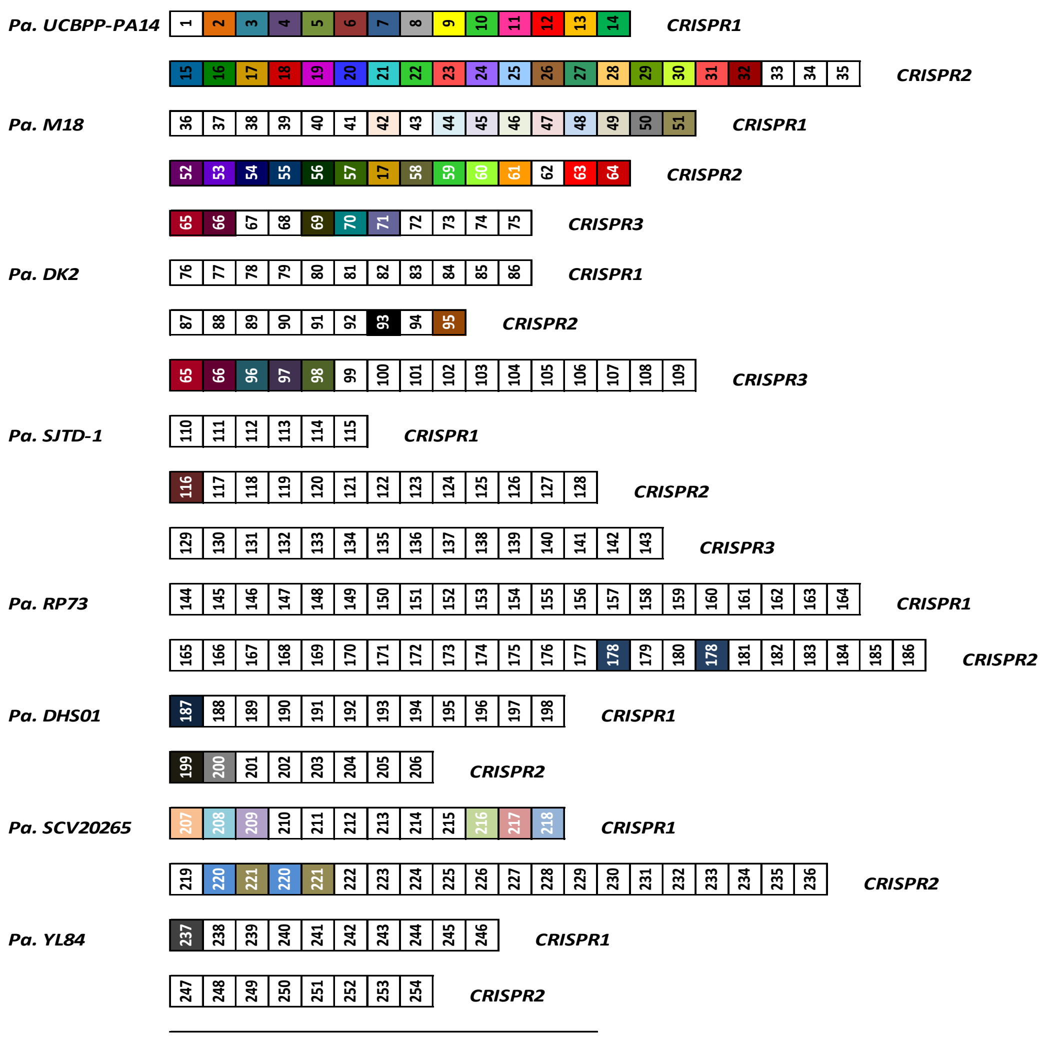

Figure S1. Diversity of spacer sequences. Spacers are represented by boxes; direct repeats (DR) are not included. The same number and color represents the same spacer. The white color represents a single spacer. The abbreviations *Pa, Pp, Ps, Pc, Pm, Pps, Pal, Pb, Ppa*, and *Pci* represent the abbreviations for *Pseudomonas aeruginosa, putida, stutzeri, chlororaphis, mendocina, pseudoalcaligenes, alcaliphila, balearica, parafulva,* and *citronellolis,* respectively.

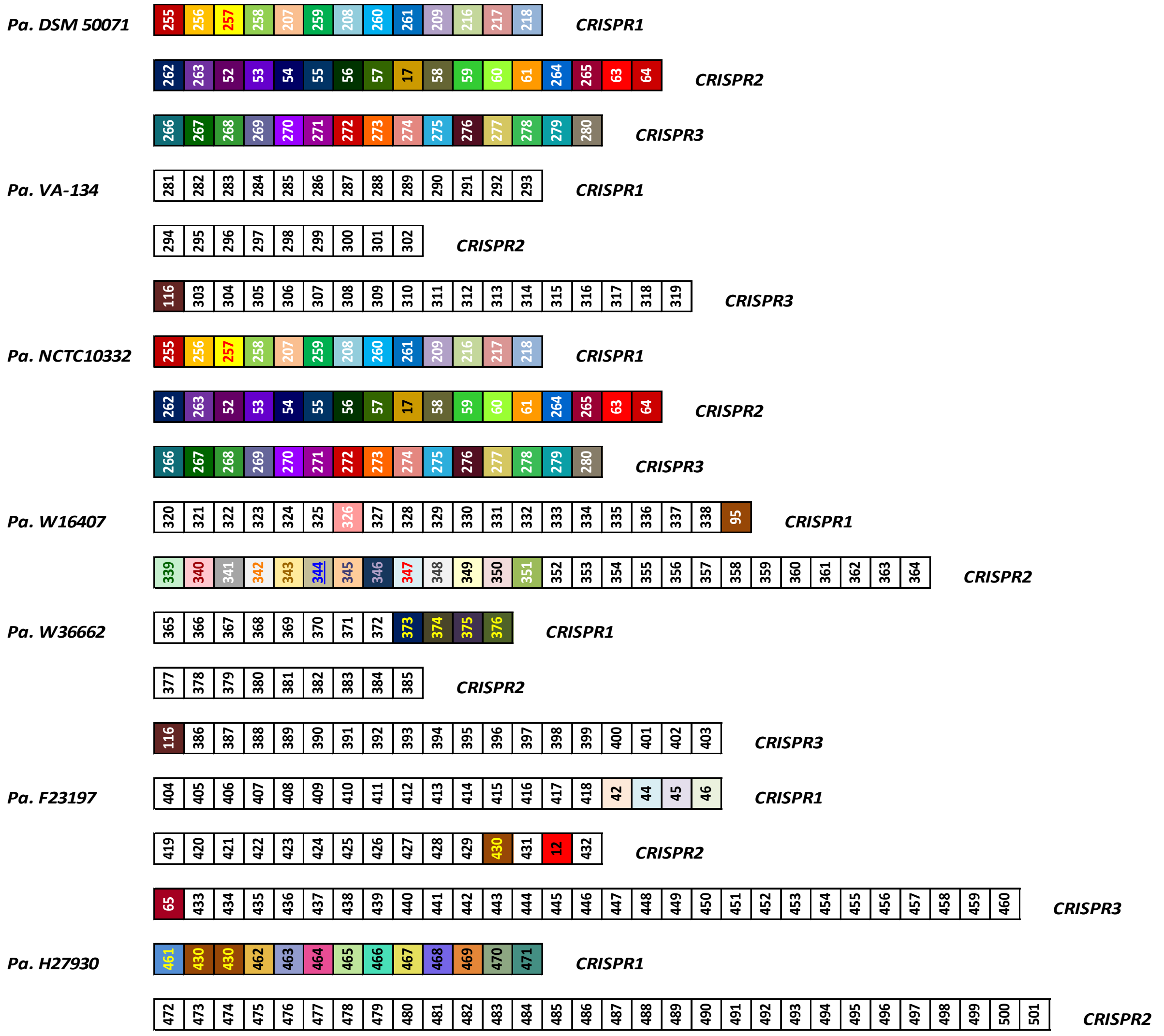

Figure S1. Diversity of spacer sequences. Spacers are represented by boxes; direct repeats (DR) are not included. The same number and color represents the same spacer. The white color represents a single spacer. The abbreviations *Pa, Pp, Ps, Pc, Pm, Pps, Pal, Pb, Ppa,* and *Pci* represent the abbreviations for *Pseudomonas aeruginosa, putida, stutzeri, chlororaphis, mendocina, pseudoalcaligenes, alcaliphila, balearica, parafulva,* and *citronellolis,* respectively.

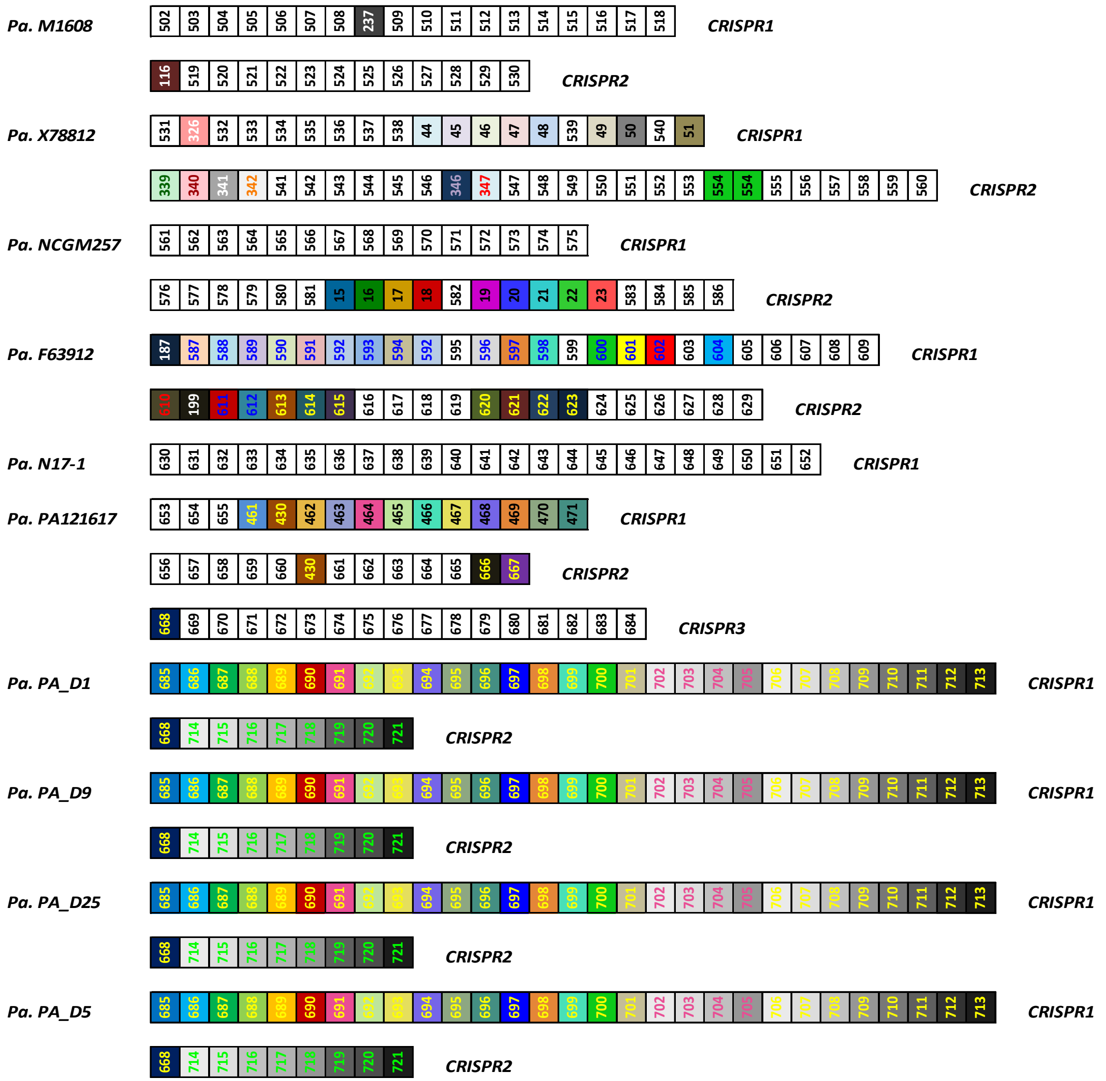

Figure S1. Diversity of spacer sequences. Spacers are represented by boxes; direct repeats (DR) are not included. The same number and color represents the same spacer. The white color represents a single spacer. The abbreviations *Pa, Pp, Ps, Pc, Pm, Pps, Pal, Pb, Ppa,* and *Pci* represent the abbreviations for *Pseudomonas aeruginosa, putida, stutzeri, chlororaphis, mendocina, pseudoalcaligenes, alcaliphila, balearica, parafulva,* and *citronellolis,* respectively.

Cont.

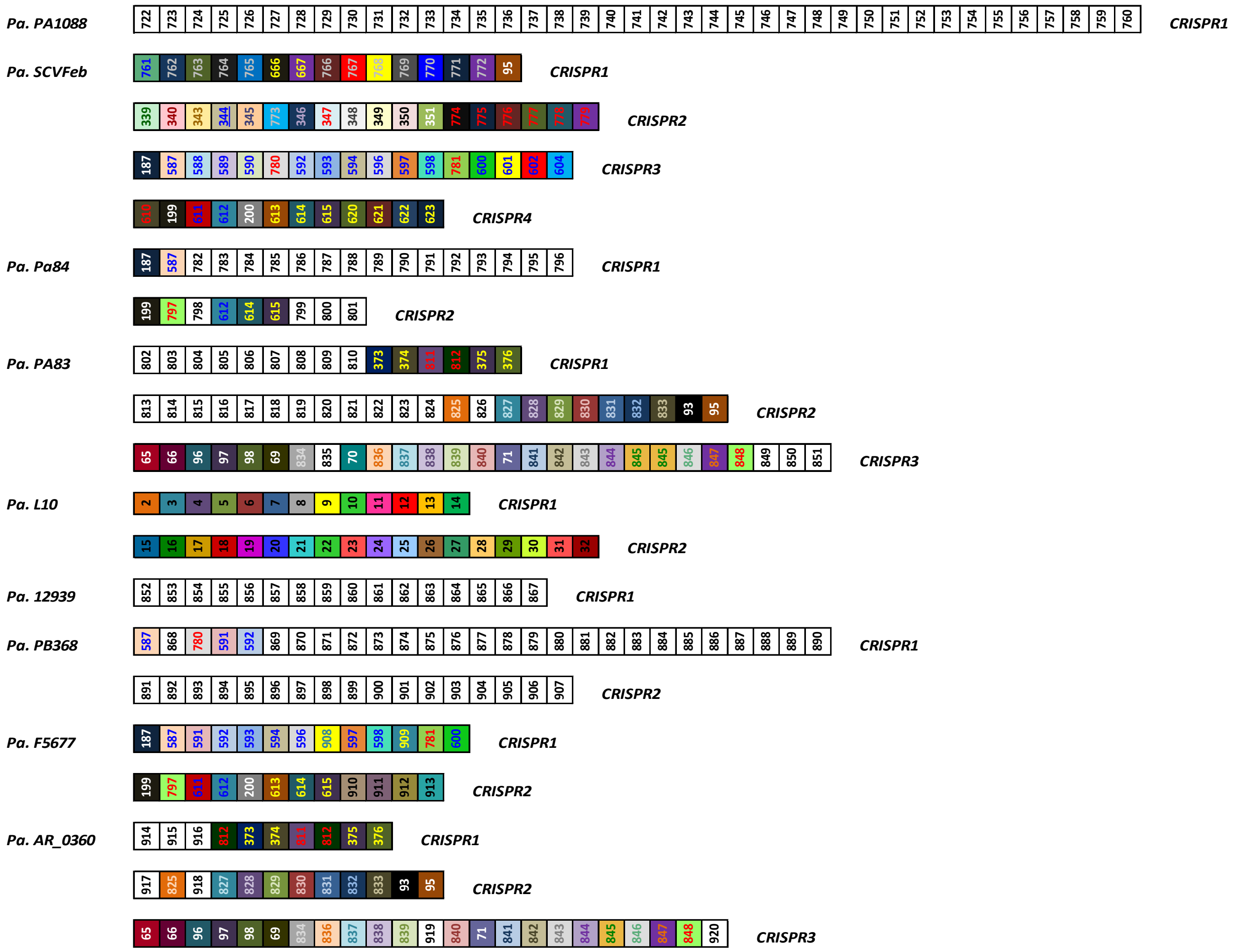

Figure S1. Diversity of spacer sequences. Spacers are represented by boxes; direct repeats (DR) are not included. The same number and color represent the same spacer. The white color represents a single spacer. The abbreviations *Pa, Pp, Ps, Pc, Pm, Pps, Pal, Pb, Ppa,* and *Pci* represent the abbreviations for *Pseudomonas aeruginosa, putida, stutzeri, chlororaphis, mendocina, pseudoalcaligenes, alcaliphila, balearica, parafulva,* and *citronellolis*, respectively.

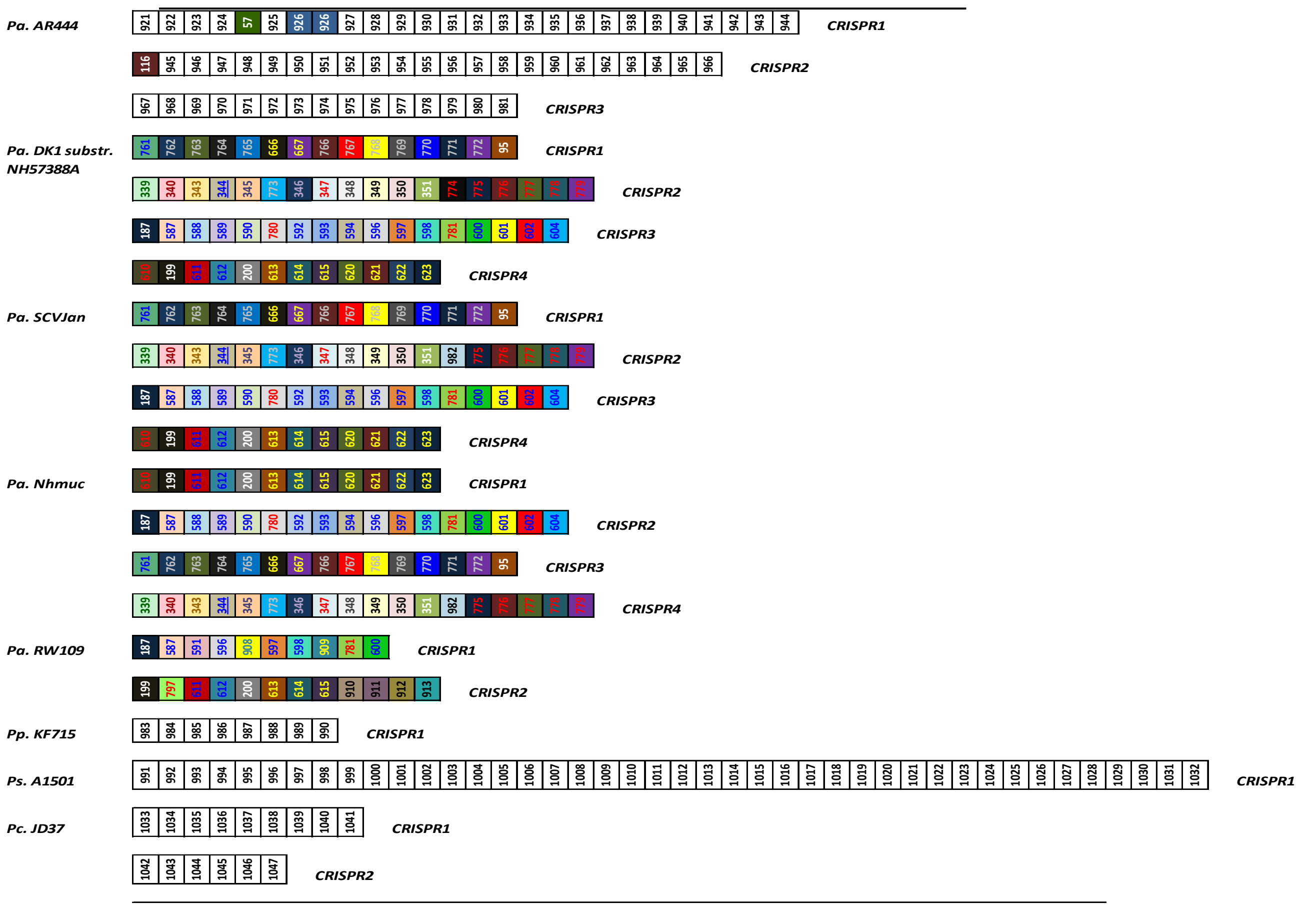

Figure S1. Diversity of spacer sequences. Spacers are represented by boxes; direct repeats (DR) are not included. The same number and color represents the same spacer. The white color represents a single spacer. The abbreviations *Pa, Pp, Ps, Pc, Pm, Pps, Pal, Pb, Ppa,* and *Pci* represent the abbreviations for *Pseudomonas aeruginosa, putida, stutzeri, chlororaphis, mendocina, pseudoalcaligenes, alcaliphila, balearica, parafulva,* and *citronellolis*, respectively.

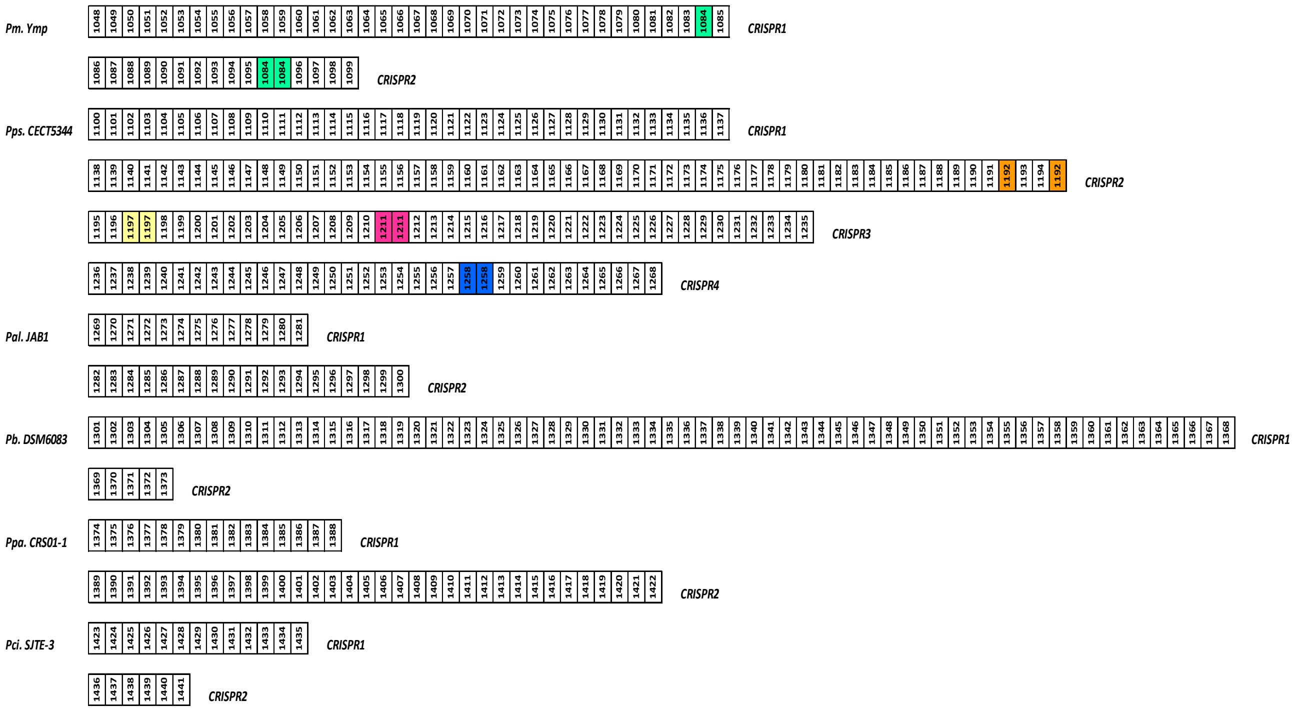

Figure S1. Diversity of spacer sequences. Spacers are represented by boxes; direct repeats (DR) are not included. The same number and color represents the same spacer. The white color represents a single spacer. The abbreviations Pa, Pp, Ps, Pc, Pm, Pps, Pal, Pb, Ppa, and Pci represent the abbreviations for Pseudomonas aeruginosa, putida, stutzeri, chlororaphis, mendocina, pseudoalcaligenes, alcaliphila, balearica, parafulva, and citronellolis, respectively.
